# Identification of sex-specific biomarkers related to programmed cell death and analysis of immune cells in ankylosing spondylitis

**DOI:** 10.1038/s41598-024-65745-3

**Published:** 2024-07-04

**Authors:** Tiantian Dong, Xuhao Li, Wenyan Yu, Yuanxiang Liu, Jiguo Yang

**Affiliations:** 1 Center for External Treatment of Traditional Chinese Medicine, Affiliated Hospital of Shandong University of Traditional Chinese Medicine, Jinan, People’s Republic of China; 2 School of Acupuncture-Moxibusion and Tuina, Shandong University of Traditional Chinese Medicine, Jinan, People’s Republic of China; 3 Department of Neurology, Affiliated Hospital of Shandong University of Traditional Chinese Medicine, Jinan, People’s Republic of China

**Keywords:** Autoimmunity, Immunological disorders, Innate immune cells

## Abstract

Ankylosing spondylitis (AS) stands as a persistent inflammatory ailment predominantly impacting the axial skeleton, with the immune system and inflammation intricately entwined in its pathogenesis. This study endeavors to elucidate gender-specific patterns in immune cell infiltration and diverse forms of cell demise within the AS milieu. The aim is to refine the diagnosis and treatment of gender-specific AS patients, thereby advancing patient outcomes. In the pursuit of our investigation, two datasets (GSE25101 and GSE73754) pertinent to ankylosing spondylitis (AS) were meticulously collected and normalized from the GEO database. Employing the CIBERSORT algorithm, we conducted a comprehensive analysis of immune cell infiltration across distinct demographic groups and genders. Subsequently, we discerned differentially expressed genes (DEGs) associated with various cell death modalities in AS patients and their healthy counterparts. Our focus extended specifically to ferroptosis-related DEGs (FRDEGs), cuproptosis-related DEGs (CRDEGs), anoikis-related DEGs (ARDEGs), autophagy-related DEGs (AURDEGs), and pyroptosis-related DEGs (PRDEGs). Further scrutiny involved discerning disparities in these DEGs between AS patients and healthy controls, as well as disparities between male and female patients. Leveraging machine learning (ML) methodologies, we formulated disease prediction models employing cell death-related DEGs (CDRDEGs) and identified biomarkers intertwined with cell death in AS. Relative to healthy controls, a myriad of differentially expressed genes (DEGs) linked to cell death surfaced in AS patients. Among AS patients, 82 FRDEGs, 29 CRDEGs, 54 AURDEGs, 21 ARDEGs, and 74 PRDEGs were identified. In male AS patients, these numbers were 78, 33, 55, 24, and 94, respectively. Female AS patients exhibited 66, 41, 40, 17, and 82 DEGs in the corresponding categories. Additionally, 36 FRDEGs, 14 CRDEGs, 19 AURDEGs, 10 ARDEGs, and 36 PRDEGs exhibited differential expression between male and female AS patients. Employing machine learning techniques, LASSO, RF, and SVM-RFE were employed to discern key DEGs related to cell death (CDRDDEGs). The six pivotal CDRDDEGs in AS patients, healthy controls, were identified as CLIC4, BIRC2, MATK, PKN2, SLC25A5, and EDEM1. For male AS patients, the three crucial CDRDDEGs were EDEM1, MAP3K11, and TRIM21, whereas for female AS patients, COX7B, PEX2, and RHEB took precedence. Furthermore, the trio of DDX3X, CAPNS1, and TMSB4Y emerged as the key CDRDDEGs distinguishing between male and female AS patients. In the realm of immune correlation, the immune infiltration abundance in female patients mirrored that of healthy controls. Notably, key genes exhibited a positive correlation with T-cell CD4 memory activation when comparing male and female patient samples. This study engenders a more profound comprehension of the molecular underpinnings governing immune cell infiltration and cell death in ankylosing spondylitis (AS). Furthermore, the discernment of gender-specific disparities among AS patients underscores the clinical significance of these findings. By identifying DEGs associated with diverse cell death modalities, this study proffers invaluable insights into potential clinical targets for AS patients, taking cognizance of gender-specific nuances. The identification of gender-specific biological targets lays the groundwork for the development of tailored diagnostic and therapeutic strategies, heralding a pivotal step toward personalized care for AS patients.

## Introduction

Ankylosing spondylitis (AS) stands as a chronic inflammatory ailment predominantly impacting the axial skeleton, inducing damage to sacroiliac and spinal joints, ultimately leading to spine fusion and rigidity, often colloquially termed "bamboo spine"^1^. The prognosis of AS varies considerably among individuals, with well-established gender-related differences in incidence and severity. The prevalence of AS ranges from 0.1% to 2%, with a higher occurrence in men, presenting a male-to-female ratio of approximately 5:1, and a typical onset age between 20 to 30 years^2,3^. Research indicates a more severe progression of spinal imaging in male patients with axial spondyloarthritis (axSpA), making them three times more prone to such advancement^4^. Understanding the intricate mechanisms behind these gender-specific differences and various forms of cell death associated with AS holds promise for unveiling new therapeutic targets, potentially tailoring treatments to the nuanced needs of AS patients, considering gender-related variations.

In recent years, programmed cell death (PCD) has garnered significant attention as a diagnostic biomarker and therapeutic target in various diseases^5^. Distinct types of PCD, such as ferroptosis, anoikis, pyroptosis, autophagy, and cuproptosis, represent orchestrated processes governed by specific genes to maintain cellular homeostasis and eliminate abnormal cells^6^. A recent study highlights the association between programmed cell death 1 (PDCD1) gene polymorphism and ankylosing spondylitis in the Korean population^7^.

Ferroptosis, a non-apoptotic form of cell death, involves iron overload, glutathione deficiency, and the inactivation of glutathione peroxidase 4 (GPX4)^8^. Genes like DDIT3 and HSPB1 are suggested as potential AS biomarkers linked to ferroptotic cell death^9^. Oxidative stress and mitochondrial dysfunction in AS are indicative of ferroptosis, supported by documented iron accumulation, lipid peroxidation, and mitochondrial dysfunction in ankylosing spondylitis tissues or cells^10^.

Anoikis serves as a mechanism employed by organisms to safeguard themselves by hindering detached cells from reattaching to abnormal substrates^11^. Despite its distinctive definition, anoikis fundamentally represents an apoptotic process^11^. The participation of Natural Killer (NK)-derived cytokines, along with their cytotoxic function in inducing apoptosis, assumes a pivotal role in the regulation of immune responses and potentially contributes to the pathogenesis of ankylosing spondylitis^12^.

Previous investigations have substantiated that individual with ankylosing spondylitis manifest significantly elevated levels of serum copper and ceruloplasmin, particularly in severe instances^13^. The surge in serum copper levels may be ascribed to a concomitant increase in cyanin, indicative of a non-specific inflammatory response. Subsequent research has unveiled heightened levels of serum copper and ceruloplasmin in ankylosing spondylitis patients, correlating with inflammatory activity^13,14^. Simultaneously, reports indicate the utility of serum copper as a diagnostic indicator for ankylosing spondylitis. Given the indispensability of copper for diverse immune functions, it becomes imperative to scrutinize the potential impact of CRGs on immune function in the development of Ankylosing Spondylitis (AS). However, pertinent literature on this subject remains scarce^15,16^.

The autophagic process represents a finely regulated catabolic mechanism that degrades cellular components and provides energy substrates during instances of nutrient deprivation and metabolic stress^17^. Studies have elucidated the role of autophagy signaling pathways, such as PKA/mTOR/ULK1, in promoting osteogenic differentiation, thereby implicating potential implications for the treatment of osteoporotic diseases^18^. Given its association with IL-23 production and secretion in ankylosing spondylitis, autophagy emerges as a noteworthy immune factor linked to AS^19^. Research suggests that the etiology of ankylosing spondylitis may be linked to impaired autophagy, with the progression of this condition in humans partially attributed to excessive fibroblast proliferation^20,21^.

Pyroptosis, characterized by cell membrane destruction and the release of cytokines promoting inflammatory and immune responses^22^, has been linked to AS patients through the implication of the NLRP3 inflammasome in pyroptotic cell death. The increased expression of NLRP3 and caspase-1 contributes to inflammatory damage, potentially influencing the pathological progression of AS^23^. Inflammasomes, activating caspase-1 and initiating pro-inflammatory cell death (pyroptosis), are implicated in the pathogenesis of AS^24,25^.

In summary, while programmed cell death processes, particularly pyroptosis, have been extensively explored in the context of AS, especially in the realms of inflammation and immunity, associations with iron death and autophagy have also been established. However, research progress in cuproptosis and anoikis remains comparatively limited. Presently, numerous investigations focus on biomarkers associated with programmed cell death in ankylosing spondylitis. Yet, there exists a scarcity of studies examining the correlation between sex-specific programmed cell death and ankylosing spondylitis. Moreover, existing research predominantly pertains to a singular form of programmed cell death, thus lacking comprehensive coverage.

**Figure 1:**
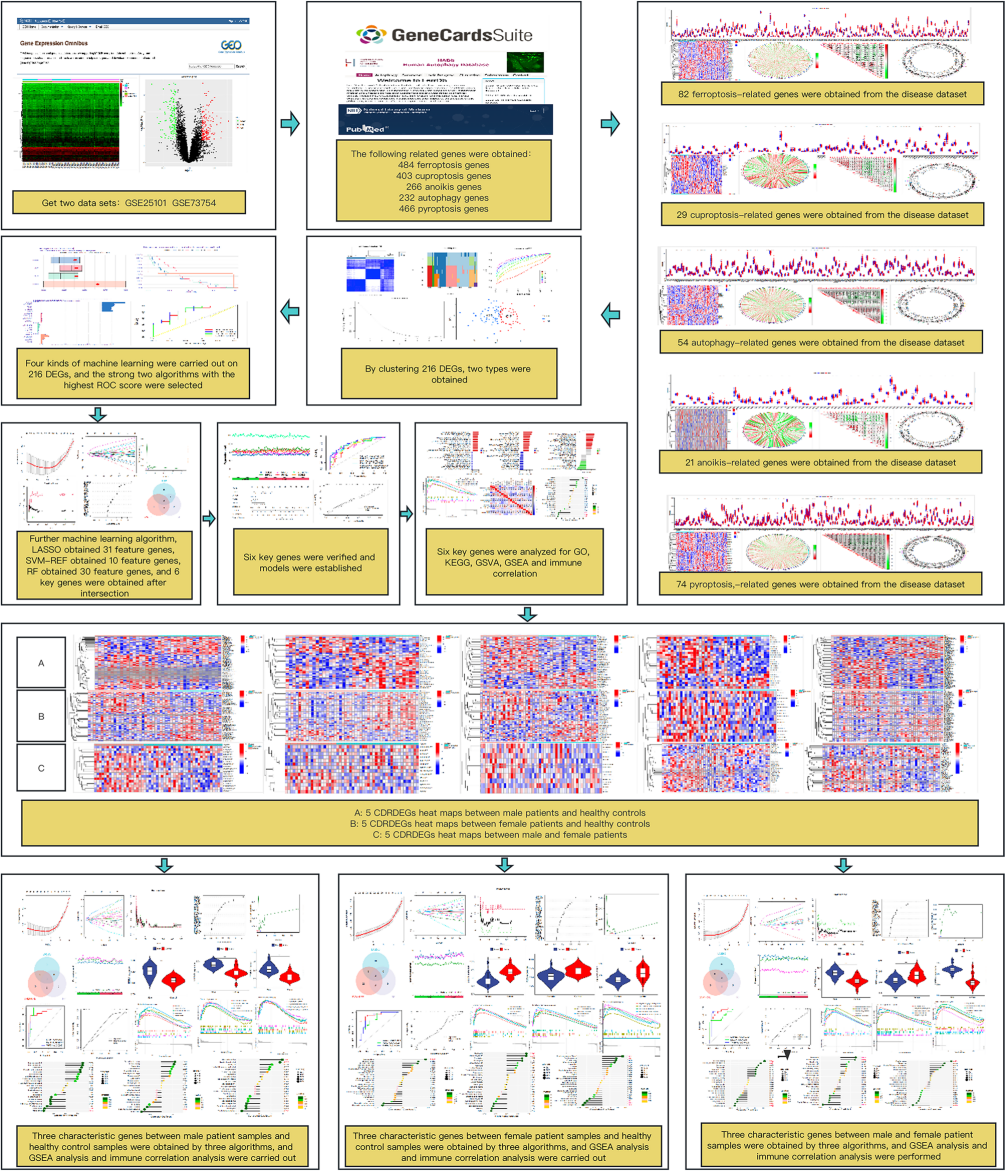
Schematic representation of the study framework.

In this study, we gathered cell death-related DEGs (CDRDEGs), encompassing ferroptosis-related genes (FRGS), pyroptosis-related genes (PRGS), autophagy-related genes (AURGS), cuproptosis-related genes (CRGS), and anoikis-related genes (ARGS). Transcriptome data were obtained from a public database for analysis. We selected differential genes for the five types of cell death-related genes from a dataset comprising male and female AS patient groups and healthy controls collected in this study. Through machine learning, we identified characteristic biomarkers and investigated their association with immune infiltration. This insight can be beneficial for both women and men in future clinical studies aimed at preventing and treating AS. Figure 1 provides information on the dataset and workflow for this study.

## Materials and methods

The study employed a meticulously devised search strategy and stringent inclusion criteria. The search protocol involved querying the GEO database specifically with the keyword "ankylosing spondylitis", with a refined focus on the "series" and "Homo sapiens" categories.

Inclusion criteria: the following criteria were systematically applied to discern and select pertinent datasets:

- The database must encompass individuals diagnosed with ankylosing spondylitis (AS) aged 18 years or above, along with a corresponding cohort of healthy controls.
- Each identified group, comprising AS patients and healthy controls, necessitated a minimum representation of three individuals.

The essential criterion for inclusion dictated that the dataset should furnish comprehensive mRNA expression data derived from whole blood.

Selected datasets: two distinct data series, denoted as GSE25101 and GSE73754, impeccably satisfied all predetermined inclusion criteria (refer to Table 1). These datasets were procured from the National Center for Biotechnology Information (NCBI) gene expression library. In the case of GSE73754, 52 samples from individuals with AS and 20 samples from healthy subjects were meticulously generated employing the GPL10558 Illumina HumanHT-12 V4.0 expression beadchip. Similarly, the GSE25101 dataset, comprising 16 AS samples and 16 normal samples, was derived utilizing the Illumina HumanHT-12 V3.0 expression beadchip. Both datasets, meticulously curated from the NCBI gene expression library, provide comprehensive gene expression matrices poised for subsequent in-depth analysis in conjunction with ankylosing spondylitis.

**Table 1:** Description of the data used in this study.

Accession	Platform	Control	AS	Male	Female	Total	Type
GSE73754	GPL10558	20	52	20	32	72	Whole blood samples
GSE25101	GPL6947	16	16	Not distinguish	Not distinguish	32	Whole blood samples

Table 1 shows the details of the selected dataset.

The Affy preprocessing procedure was conducted on the two primary datasets using R (version 4.2.1). The integration of both datasets involved the application of the 'sva' batch effect removal and the 'limma' package, encompassing background calibration, normalization, and log2 transformation. The consolidation of these datasets was achieved through a meticulous process. For the analysis and visualization of data and graphs in this manuscript, R version 4.2.1 was employed.

### Obtainment of 5 cell death-related DEGs (CDRDEGs)

Our focus centered on delineating five distinct classes of Cell Death-Related Differentially Expressed Genes (CDRDEGs) and assembling their respective associated genes. These genes were meticulously sourced from various repositories based on their pertinence to diverse facets of cell death. To discern genes associated with anoikis, the GeneCards database (https://www.genecards.org) was leveraged, employing a stringent screening criterion of a relevance score surpassing 0.4. Autophagy-related genes were curated from the human Autophagy Database (HADb; http://www.autophagy.lu). For genes implicated in iron death and scorch death, we referenced the iron death database (FerrDb; http://www.zhounan.org/ferrdb) in conjunction with published literature. Genes pertaining to copper ptosis were exclusively gleaned from relevant published literature. Our comprehensive approach encompassed the isolation of these genes from both disease and control samples, as well as male and female samples, facilitating an exploration of potential variances or associations with cell death.

In our pursuit of disease/control and male/female Differentially Expressed Genes (DEGs), we employed R's "limma" package, implementing an adjustment threshold of "adjust-p = 0.05". These DEGs were subsequently classified as FRDEGs, PRDEGs, ARDEGs, CRDEGs, and AURDEGs. The "ggpubr" and "pheatmap" packages in R were utilized for the generation of gene box line plots and co-expression networks. To elucidate the genomic positions of the DEGs on human chromosomes, the "RCircos" package in R was harnessed. Additionally, we explored the interrelations between DEGs and immune cells through comprehensive enrichment analyses. These methodological strategies were adeptly implemented to alleviate redundancy and furnish a succinct overview of our approach towards DEG identification, gene expression visualization, genomic location mapping, and functional interactions with immune cells.

### A machine learning paradigm for identifying CDRDEG biomarkers

Expression data of FRDEGs, PRDEGs, ARDEGs, CRDEGs, and AURDEGs were incorporated into the DEGs samples. Random Forest (RF), Support Vector Machine (SVM), Extreme Gradient Boosting (XGB), Generalized Linear Model (GLM), Least Absolute Shrinkage and Selection Operator (LASSO), and Support Vector Machine Recursive Feature Elimination (SVM-RFE) constituted the six machine learning models, with samples randomly divided into training (70%) and test (30%) sets. These models were instrumental in screening core genes, illustrating model residuals, and discerning disease biomarkers from CDRDEGs. Machine learning models and Receiver Operating Characteristic (ROC) curves were employed for these purposes, exemplifying a comprehensive and rigorous approach towards identifying biomarkers associated with cell death.

### Consensus clustering

Within the framework of consensus clustering, the determination of the number of unsupervised clusters in a given dataset assumes significance. The GSE73754 and GSE25101 datasets were instrumental in this study to categorize samples of AS patients into discernible ICI clusters. Employing the R package Consensus ClusterPlus, our analysis underwent 100 iterations for heightened precision and reproducibility. The outcomes were meticulously visualized using the 'pheatmap' package in R, encompassing the consistency matrix, Cumulative Distribution Function (CDF) diagram, relative change in the area under the CDF curve, and trajectory diagram. These visual representations, coupled with the results derived from the consensus clustering analysis, facilitate the identification of the optimal number of clusters for AS patient samples.

### Functional enrichment and immune infiltration analysis

The analytical toolkit comprised the Cluster Profiler package and enrichplot package in R for dissecting functional enrichment within the dataset. A comprehensive exploration, including Kyoto Encyclopedia of Genes and Genomes (KEGG)^26–28^ and Gene Ontology (GO) analyses, was conducted. Further enhancement of the analysis involved a single-sample gene set enrichment analysis (ssGSEA). The deconvolution of the expression matrix of human immune cell subtypes was achieved using CIBERSORT in R. Calculations based on 100 permutations elucidated the proportions of 22 immune cells, with results seamlessly visualized using R. The immune correlation of pivotal genes and their potential regulatory influence on immune cells was intricately computed using the corrplot package in R. In essence, our study aimed at discerning disparities in immune cell infiltration between ankylosing spondylitis patients and healthy controls, as well as variations between male and female cohorts. This objective was accomplished through the implementation of the CIBERSORT algorithm, complemented by gender-specific evaluations.

### Gene set variation analysis (GSVA)

The application of Gene Set Variation Analysis (GSVA), a non-parametric unsupervised analytical approach, facilitates the evaluation of gene set enrichment outcomes in microarrays and transcriptomes. Within this analytical framework, the R software packages "GSVA" and "GSEABase" were deployed to scrutinize the variability within the dataset. The initial step involved the extraction of the gene set specific to the disease group by excluding samples from the control group. Subsequently, the gene set associated with this group of diseases underwent adjustment and assessment through the GSVA technique. The primary objective was the identification of the top ten pathways displaying significant enrichment. The 'limma' software package was employed for the analysis and comparison of variations in GSVA scores across diverse subtypes. Limma, renowned for detecting statistically significant variations in expression levels between groups, played a pivotal role in this investigation. To summarize, for the evaluation of gene set enrichment in microarray data, this study harnessed the GSVA and GSEA Base packages within the R software. The GSVA method was instrumental in extracting and evaluating the gene set specific to the disease group, while the Limma package facilitated the comparison of GSVA scores across various subtypes.

### Statistical analysis

Within this study, statistical analyses were conducted utilizing R software, specifically version 4.2.1, along with pertinent supporting packages. The principal objective was the exploration of associations and correlations among different sets of continuous variables.

This study encompassed two distinct groups or sets of continuous variables, subjected to rigorous statistical scrutiny. These groups likely represent specific characteristics or measurements relevant to the research focus. Precise definition and elaboration on these groups are imperative to enhance reader comprehension. To assess the association between the two sets of continuous variables, the nonparametric Wilcoxon rank sum test was applied. This test, suitable for comparing distributions in the absence of normality assumptions, provided insights into significant differences between the two groups.

Spearman correlation analysis was employed to ascertain the correlation coefficient between variables. In contrast to Pearson correlation, Spearman correlation does not assume a linear relationship and is apt for assessing monotonic associations. Providing clarity on the specific variables involved in the correlation analysis and their biological or clinical relevance would enhance the interpretability of findings. Statistical significance was determined using a predefined significance level of p < 0.05, a critical threshold for establishing the credibility of observed associations and correlations.

The 'rms' package was utilized for merging characteristic genes and constructing a nomogram. It is valuable to elucidate the characteristics of these genes and their significance in the research context. Details on nomogram development methodology and its application to the research question should be provided. The calibration curve assessed the precision of the nomogram by comparing predicted outcomes with actual observed outcomes. Decision curve analysis evaluated the clinical usefulness of the nomogram. A comprehensive explanation of these evaluation metrics, their relevance to the study, and interpretation of results is necessary for reader understanding. To enhance result interpretability, a Principal Component Analysis (PCA) plot visualization was generated using the ggplot2 package. Clearly stating the purpose of generating this plot, the variables represented, and insights gained from the visualization would strengthen the overall presentation of findings.

## Results

### Ferroptosis-related genes (FRGs)

**Figure 2:**
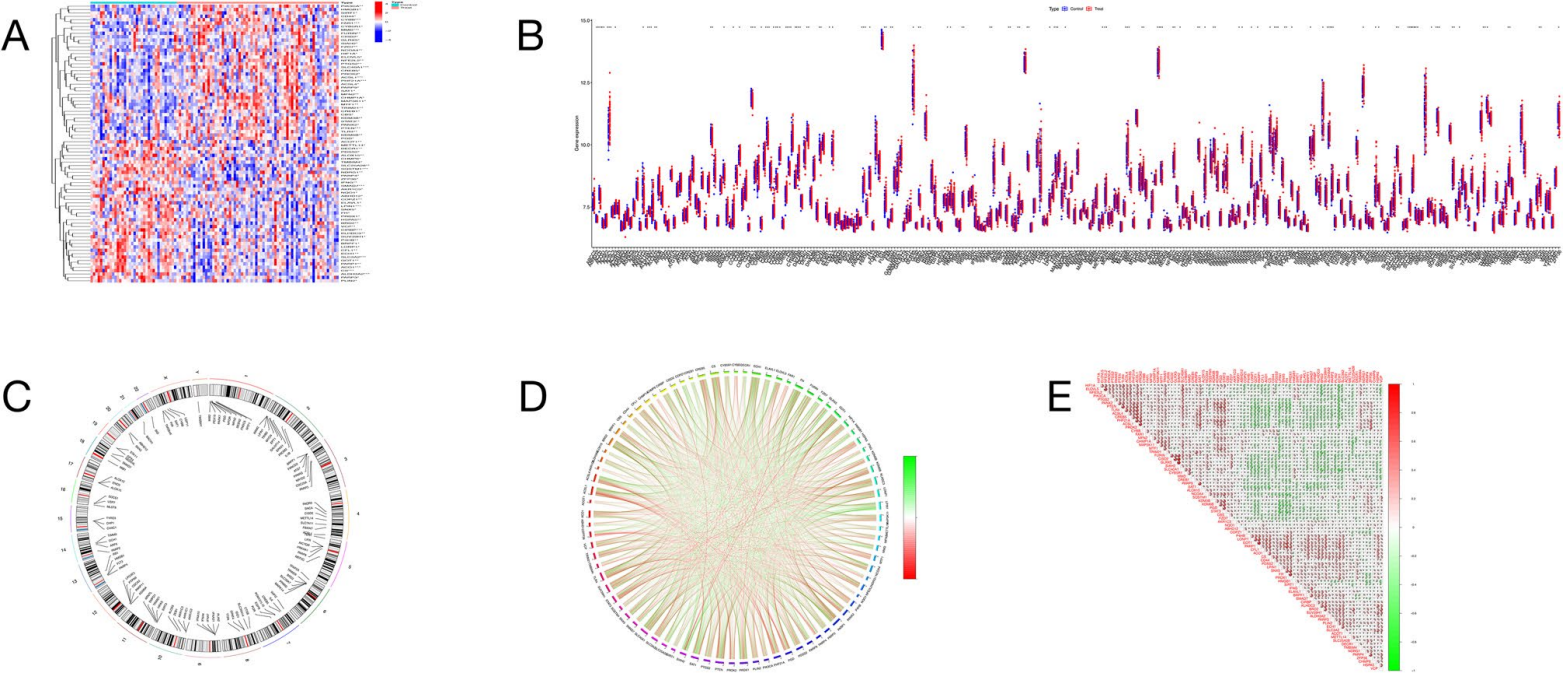
Identification of biomarkers of ferroptosis-related DEGs (FRDEGs) between disease and healthy samples. (**A**) Heat map comparing the disease and healthy samples. (**B**) Violin plots illustrating the contrast between the disease and healthy samples. (**C**) Chromosomal loci distribution of FRDEGs in the disease and healthy samples. (**D**) Association map of FRDEGs between the disease and healthy groups. (**E**) Correlation map depicting the relationship among FRDEGs in the disease and healthy groups. (**A**) In the (**A**), the horizontal axis is the classification, where the light green is the healthy control group, the light red is the disease group, and the vertical axis is DEGs (FRDEGs). The darker the red is, the higher the up-regulated gene expression, and the darker the blue is, the higher the down-regulated gene expression. (**B**) In the chart FIG2B, the horizontal axis is DEGs (FRDEGs), and the vertical axis is gene expression. In the type, blue represents the healthy control group, and red represents the disease group. The asterisk at the top of the chart represents the difference between the two groups, asterisk represents p < 0.05 in the comparison between the two groups, double asterisk represents p < 0.01 in the comparison between the two groups, triple asterisk represents p < 0.001 in the comparison between the two groups. (**C**) The inside of the circle is DEGs (FRDEGs), and the point corresponding to the line is the chromosome position of the gene. (**D**) The periphery of the circle is DEGs (FRDEGs), and the internal connection of the circle is the correlation between the two genes. The reder the color is, the stronger the positive correlation is. The greener the color is, the stronger the negative correlation is. (**E**) The horizontal axis of the picture and the oblique axis of the triangle are DEGs (FRDEGs). The intermediate correlation is expressed by fan-shaped area and color. The larger the fan-shaped area is, the stronger the correlation is. The smaller the area is, the smaller the correlation is. The reder the color is, the stronger the positive correlation is. The greener the color is, the stronger the negative correlation is.

Upon completion of the FRGS selection process, a total of 82 FRDEGs were identified between the alternative splicing (AS) sample and the control sample. These FRDEGs serve as crucial indicators of differential gene expression in our study. To provide a clearer understanding of the identified FRDEGs, we present their detailed analysis and characterization in the subsequent sections. To highlight the key results, the heat maps and violin charts of the 82 FRDEGs are meticulously presented in Fig. 2A, B. These visual representations offer a comprehensive view of the expression patterns and distribution of these genes, emphasizing the significance of their differential expression in the context of our study. Figure 2C elucidates the chromosome distribution of the identified FRDEGs.

This analysis provides insights into the genomic locations of these genes, potentially revealing underlying genomic trends or biases. The spatial arrangement of FRDEGs across chromosomes is essential in understanding the genetic context of their expression differences. Figure 2D, E delve into the correlation among the FRDEGs. The correlation analysis sheds light on potential interactions and co-regulations among these genes. This information is critical in unraveling the complex regulatory networks that may underlie the observed differences in gene expression between the AS sample and the control sample.

### Cuproptosis-related genes (CRGs)

We expanded our focus to cuproptosis-related genes (CRGs) to unravel their role in the observed phenotypic differences between the AS sample and the control sample. Through rigorous analysis, we identified a set of 29 CRGs, referred to as Cuproptosis-Related Differentially Expressed Genes (CRDEGs). Within this subset of CRDEGs, specific genes exhibited significant dysregulation. To highlight these key findings, we have included heat maps (Fig. S1A) and violin charts (Fig. S1B) to visually represent the expression patterns. These graphical representations serve to emphasize the magnitude and direction of expression changes in the identified genes.

To gain further insights into the genomic context and relationships among CRDEGs, we employed additional analyses. The chromosome distribution of CRDEGs is illustrated in Fig. S1C, offering a spatial perspective on the genomic locations of these genes. Furthermore, correlation analyses were conducted to unveil the intricate relationships among CRDEGs. Figure S1D, E depict correlation matrices that connect the expression patterns of specific genes within the CRDEGs subset. These analyses contribute to a comprehensive understanding of how these genes interact and collectively contribute to cuproptosis.

### Autophagy-related genes (AURGs)

Upon the selection of Autophagy-Related Genes (AURGS), a comprehensive analysis revealed 54 significantly Differentially Expressed Genes (AURDEGs) between the Ankylosing Spondylitis (AS) sample and the control sample. The expression profiles of these AURDEGs were visually represented through heat maps and violin charts, providing a detailed overview of the differential expression patterns (see Fig. S2A, B). Subsequently, a chromosomal distribution analysis was conducted to gain insights into the genomic locations of the identified AURDEGs, as illustrated in Fig. S2C.

To establish a more holistic understanding, we delved into the correlation patterns among the AURDEGs. Figure S2D presents a correlation map depicting the interplay among these genes, shedding light on potential regulatory relationships. Additionally, Fig. S2E showcases a correlation network, providing a visual representation of the associations among the identified AURDEGs. These correlation analyses contribute valuable insights into the potential synergies or regulatory interactions within the autophagy-related gene network.

### Anoikis-related genes (ARGs)

In our investigation of Anoikis-related genes (ARGs), we identified a total of 21 Anoikis-Related Differentially Expressed Genes (ARDEGs) that exhibited significant dysregulation between the AS sample and the control sample. Our focus on these specific genes aimed to unravel the molecular intricacies underlying the observed phenotypic differences.

To emphasize the key findings, we present a comprehensive analysis of the ARDEGs, shedding light on their specific roles and connections. Heat maps and violin charts visually show the unique expression patterns of the identified genes (Fig. S3A, B). These graphical representations not only enhance the clarity of our results but also provide a compelling snapshot of the differential expression landscape.

Furthermore, we delved into the genomic context of ARDEGs through a chromosome distribution analysis (Fig. S3C). This visualization aids in elucidating the genomic locations of dysregulated genes, offering insights into potential chromosomal hotspots or clusters associated with the observed changes.

To establish interrelationships among ARDEGs, we conducted correlation analyses (Fig. S3D, E). These figures not only highlight the connections between individual genes but also contribute to a more holistic understanding of the regulatory networks at play. By identifying co-expression patterns, we can discern potential pathways or functional modules that may be collectively contributing to the observed differences in Anoikis sensitivity.

### Pyroptosis-related genes (PRGs) and integration of cell death-related genes (CDRDEGs)

Upon the selection of all PRGS, a total of 74 PRDEGs were identified between the AS sample and the control sample. The heat maps and violin charts illustrating their expression patterns are presented in Fig. S4A, B, respectively. The chromosomal distribution of these genes is visually depicted in Fig. S4C, while their correlation is meticulously elucidated in Fig. S4D, E.

Through a meticulous screening process of AS and control samples, a comprehensive set of genes was identified, including 82 FRDEGs, 29 CRDEGs, 54 AURDEGs, 21 ARDEGs, and the previously mentioned 74 PRDEGs. Post removal of 44 duplicate genes, a refined set of 216 CDRDEGs was derived. Notably, the distribution of these genes revealed that FRDEGs constituted 31.5% of CDRDEGs, CRDEGs accounted for 11.2%, AURDEGs represented 20.8%, ARDEGs contributed 8.1%, and PRDEGs encompassed 28.5% of the CDRDEGs. Consequently, it is evident that genes associated with ferroptosis and pyroptosis hold the predominant share among cell death-related mechanisms in the context of ankylosing spondylitis.

### Cluster analysis and machine learning

Firstly, we identified 216 genes exhibiting differential associations with five distinct forms of cell death in samples obtained from the AS patient group as opposed to those from the cohort of healthy controls. Employing a consistent clustering methodology throughout, the differentiation of gene samples was performed. Analysis of the region beneath the agreement matrix plot, CDF plot, and CDF curve, along with the trajectory plot (Fig. S5A–D), unequivocally established the optimal count of subtypes as 2. Subsequently, these subtypes were denoted as C1 and C2. PCA uncovered noteworthy distinctions between the two subtypes, as evidenced by the heat map and violin map (Fig. S5E, F–G).

Biomarker screening ensued utilizing RF, SVM, XGB, and GLM (Fig.S6A–D). Outcomes indicated that SVM and RF exhibited superior ROC efficiency and smaller residuals across both disease and healthy groups. The ROC for RF stood at 0.820, while SVM-REF achieved a ROC of 0.850. Subsequently, a deep learning approach was undertaken employing LASSO, RF, and SVM-REF. LASSO identified 31 characteristic genes, SVM-REF identified ten characteristic genes, RF identified 30 characteristic genes, and their intersection in the VENN diagram revealed six key genes (Fig. S7A–F): CLIC4, BIRC2, MATK, PKN2, SLC25A5, and EDEM1, respectively.

### Risk model and functional enrichment analysis

**Figure 3:**
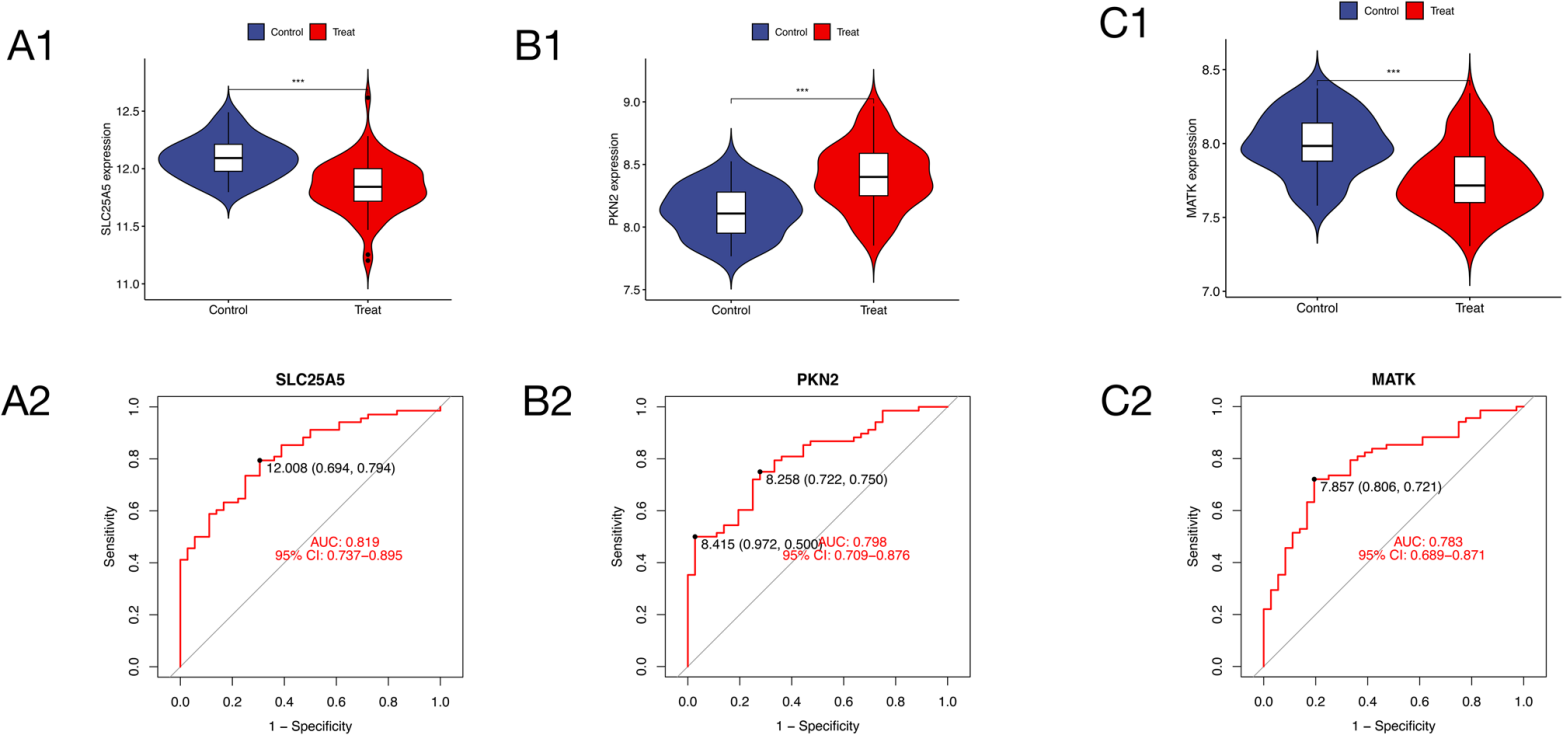
Comparison of expression changes in three key genes (SLC25A5, PKN2, and MATK) between the disease and healthy groups, along with their respective sensitivity and specificity levels. (**A1**) Expression of SLC25A5 between the disease and healthy groups. SLC25A5 was down-regulated in the disease. (**A2**) ROC curve of SLC25A5 gene, AUC = 0.819. (**B1**) Expression of PKN2 between the disease and healthy groups. PKN2 was up-regulated in the disease. (**B2**) ROC curve of PKN2 gene, AUC = 0.798. (**C1**) Expression of MATK between the disease and healthy groups. MATK was down-regulated in the disease. (**C2**) ROC curve of MATK gene, AUC = 0.783. (**A1–C1**) The horizontal axis of the picture is grouped, blue is the control group, red is the disease group, and the vertical axis is the expression of the gene. The asterisk between the two groups represents the difference between the two groups. Asterisk represents the comparison between the two groups p < 0.05, double asterisk represents the comparison between the two groups p < 0.01, triple asterisk represents the comparison between the two groups p < 0.001. (**A2–C2**) The horizontal axis of the picture is the Specificity of the gene, and the vertical axis is the sensitivity of the gene. The area under the red line in the picture represents the area value of AUC.

**Figure 4:**
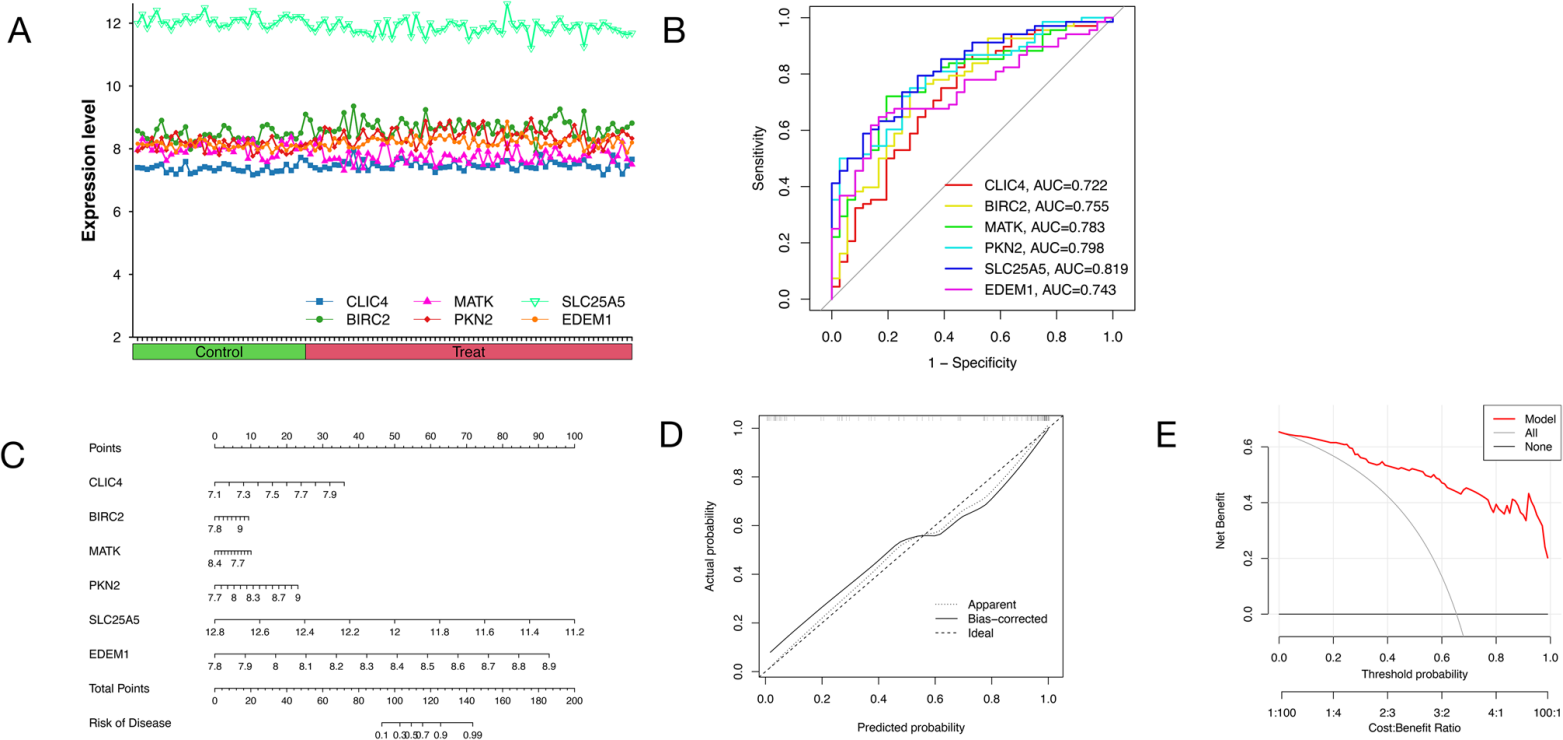
Line chart, comprehensive ROC and nomogram model of CLIC4, BIRC2, MATK, PKN2, SLC25A5 and EDEM1. (**A**) Line chart of CLIC4, BIRC2, MATK, PKN2, SLC25A5 and EDEM1. (**B**) Comprehensive ROC diagram of 6 key genes. (**C**) Nomogram risk model. (**D**) Nomogram risk model calibration curves. (**E**) Clinical advantage demonstrated by decision curve analysis. (**A**) The horizontal axis of the picture is the grouping, the green is the control group, the red is the disease group, and the vertical axis is the gene expression. The different color lines in the picture represent different genes. (**B**) The horizontal axis of the picture is the specificity of the gene, and the vertical axis is the sensitivity of the gene. Different colors represent different genes, and the area under the line represents the amount of AUC. (**C**) The horizontal axis represents the risk of disease, and the vertical axis is the risk of disease caused by each gene and the risk of disease caused by the six genes as a whole. (**D**) The horizontal axis of the image represents Predicted probability, the vertical axis represents Actual probability, the short dotted line represents Apparent, and the solid line represents Bias-corrected. The long dotted line represents deal, and the closer the short dotted line and the solid line are to the long dotted line, the higher the credibility of the model is. (**E**) The horizontal axis represents threshold probability and cost : benefit ratio, and the vertical axis represents net benefit ; the red line represents model, the gray line represents all, and the black line represents none. The closer the red line to the gray line, the higher the credibility of the model.

Examination of the comparative analysis of six pivotal genes between the cohort afflicted with AS and the healthy counterpart revealed distinct regulatory patterns. Specifically, SLC25A5 exhibited down-regulation in the AS group, evidenced by an AUC of 0.819. Conversely, PKN2 displayed up-regulation in the disease context, yielding an AUC of 0.798. MATK demonstrated down-regulation in AS, with an associated AUC of 0.783. The genes BIRC2, EDEM1, and CLIC4 exhibited up-regulation in the AS group, as denoted by AUC values of 0.755, 0.743, and 0.722, respectively (Fig. 3, Fig. S8). The collective trend and the amalgamated ROC curves for these six key genes are delineated in Fig. 4A, B.

Moreover, amalgamating these six variables into a singular entity, a nomogram (Fig. 4C) was crafted by incorporating pertinent clinical characteristics. The cumulative score, derived from the summation of individual gene scores within the nomogram, corresponded to diverse AS risk levels. The calibration curve (Fig. 4D) attested to the nomogram's precision in estimating AS prediction. Subsequently, the nomogram in Fig. 4E elucidated that individuals grappling with AS could discern their predictive risk, underscoring the robust predictive prowess of the model.

### GSEA analysis of key genes

**Figure 5:**
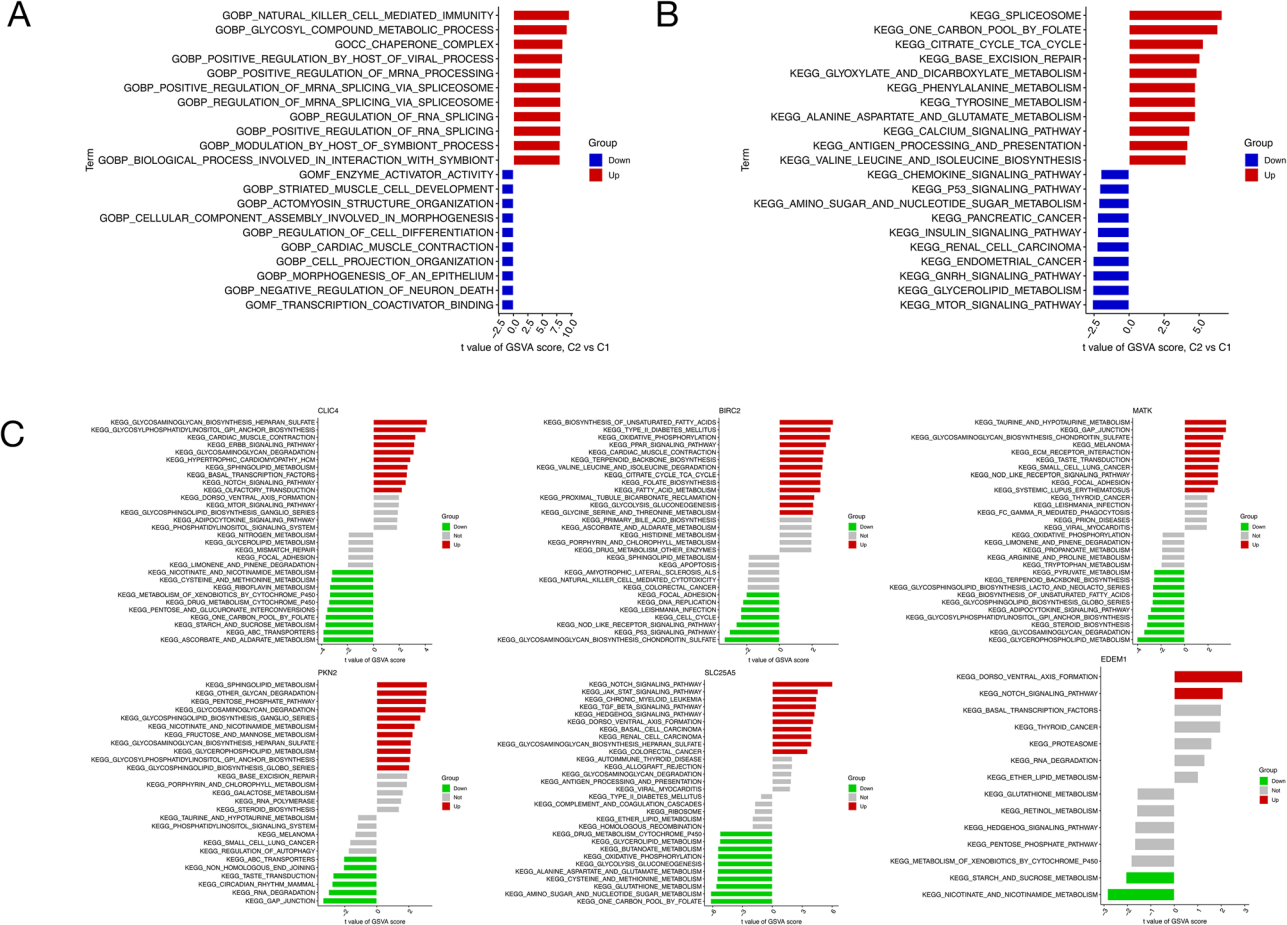
GSVA analysis of CLIC4, BIRC2, MATK, PKN2, SLC25A5 and EDEM1. (**A**) Gene Ontology (GO) histogram of CLIC4, BIRC2, MATK, PKN2, SLC25A5 and EDEM1. (**B**) Kyoto encyclopedia of genes and genomes (KEGG) histogram of CLIC4, BIRC2, MATK, PKN2, SLC25A5 and EDEM1. (**C**) GSVA of CLIC4, BIRC2, MATK, PKN2, SLC25A5, EDEM1 (note: the data presented in this figure is copyrighted to Kanehisa LABS, 'KEGG channel database'). (**A,B**) The horizontal axis represents the t value of GSVA score, and the vertical axis represents the function or pathway enriched by GO or KEGG. The groups were divided into two groups. Blue was the down-regulated gene, and red was the up-regulated gene. The longer the horizontal axis, the more functions or pathways the gene was enriched. (**C**) The horizontal axis represents the t value of GSVA score, and the vertical axis represents the pathway enriched by KEGG. Grouped into three groups, green represents the negative correlation score of the gene in the pathway, red represents the positive correlation score of the gene in the pathway, and the longer the horizontal axis represents the stronger the correlation of the pathway enriched by the gene.

Through GSVA score calculation, the top 10 pathways associated with six pivotal genes in GO and KEGG were delineated. Illustrated in Fig. 5A, B, the biological processes of natural killer cell-mediated immunity and glycosyl compound metabolic process exhibit significant up-regulation, while the molecular function of transcription coactivator binding experiences down-regulation. In the realm of KEGG, spliceosome, one-carbon pool by folate, and citrate cycle pathways demonstrate notable up-regulation, contrasting with the down-regulation observed in the mTOR signaling pathway, glycerolipid metabolism, and GnRH signaling pathway.

The GSVA analysis of the six key genes is depicted in Fig. 5C. SLC25A5 primarily participates in the up-regulation pathway of the Notch signaling pathway, while down-regulation pathways in folate, amino sugar, and nucleotide sugar metabolism contribute to the establishment of a single carbon pool. PKN2 predominantly engages in two up-regulated pathways related to sphingolipid metabolism and other glycan degradation, along with a down-regulated pathway associated with gap junction. MATK demonstrates predominant enrichment in two up-regulated pathways, namely taurine and hypotaurine metabolism and gap junction, with glycerophospholipid metabolism being a down-regulated pathway. EDEM1 exhibits significant enrichment in the up-regulation pathway of dorsoventral axis formation, alongside the down-regulation pathway of nicotinate and nicotinamide metabolism. CLIC4 is primarily enriched in two up-regulated pathways related to glycosaminoglycan biosynthesis heparan sulfate and GPI anchor biosynthesis, while concurrently manifesting down-regulation in the ascorbate and aldarate metabolism pathway. Notably, the up-regulation pathway of unsaturated fatty acid biosynthesis and the down-regulation pathway of chondroitin sulfate biosynthesis are pivotal areas of BIRC2 enrichment.

**Figure 6:**
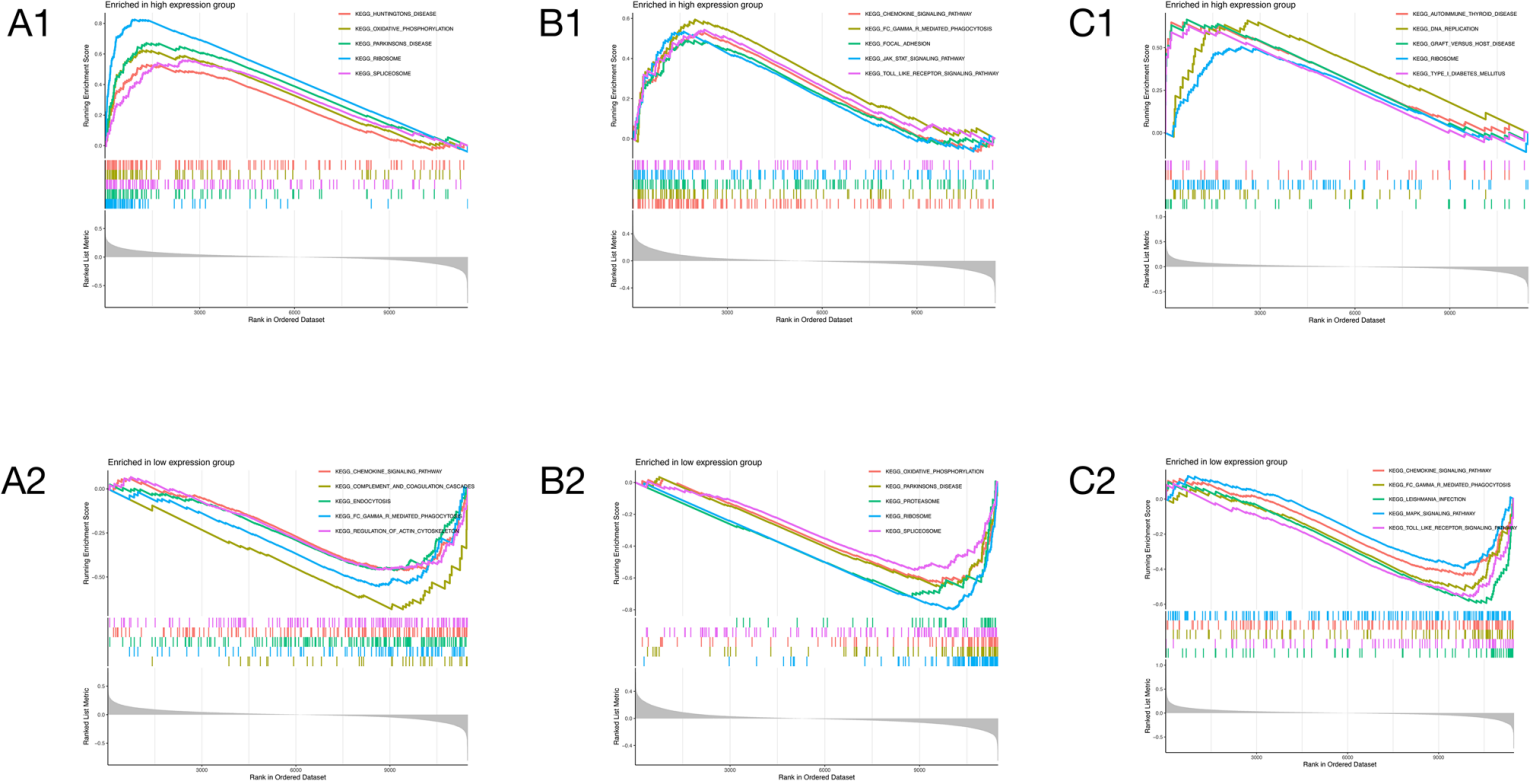
Gene set enrichment analysis (GSEA) of SLC25A5, PKN2, and MATK. (**A1**) GSEA analysis of SLC25A5 in the high expression group. (**A2**) GSEA analysis of SLC25A5 in the low expression group. (**B1**) GSEA analysis of PKN2 in the high expression group. (**B2**) GSEA analysis of PKN2 in the low expression group. (**C1**) GSEA analysis of MATK in the high expression group. (**C2**) GSEA analysis of MATK in the low expression group. (**A1–C1**) The figure is the enrichment of genes in the high expression group. The horizontal axis represents the Rank in Ordered Dataset, the vertical axis represents the Running Enrichment Score, different colors represent different KEGG pathways enriched by genes, and the upper right corner is the ranking of related pathways from top to bottom. (**A2–C2**) The picture is the enrichment of genes in the low expression group. The horizontal axis represents the Rank in Ordered Dataset, the vertical axis represents the Running Enrichment Score, different colors represent different KEGG pathways enriched by genes, and the upper right corner is the ranking of related pathways from top to bottom.

The GSEA analysis unveiled notable enrichment patterns within distinct expression groups for various genes implicated in ankylosing spondylitis. SLC25A5 demonstrated significant enrichment in huntington's disease within the high expression group, while its predominant association with the chemokine signaling pathway was evident in the low expression group (Fig. 6A1, A2). PKN2, within the high expression cohort, exhibited noteworthy enrichment in the chemokine signaling pathway, contrasting with its primary association with oxidative phosphorylation in the low expression group (Fig. 6B1, B2).

MATK, in the high expression group, demonstrated substantial enrichment in autoimmune thyroid disease, whereas in the low expression group, the chemokine signaling pathway exhibited predominant enrichment of MATK (Fig. 6C1, C2). EDEM1's enrichment profile varied between the high and low expression groups, with a predominant association with Huntington's disease and apoptosis, respectively (Fig. S9A1, A2). Similarly, CLIC4 exhibited distinct enrichments, being predominantly associated with Alzheimer's disease in the high expression group and primarily linked to acute myeloid leukemia in the low expression group (Fig. S9B1, B2).

Furthermore, BIRC2 displayed divergent enrichment patterns between the high and low expression groups, demonstrating a predominant association with the complement and coagulation cascades in the former and primarily enriching oxidative phosphorylation in the latter (FIG. S9C1, C2). These findings provide intricate insights into the nuanced regulatory roles of these genes in ankylosing spondylitis, shedding light on potential pathways and processes implicated in the pathogenesis of this condition.

### CDRDEGs biomarkers in male and female patients

The acquired AS samples were categorically stratified into three distinct groups: male samples in comparison to control samples, female samples in comparison to control samples, and a comparative analysis between male and female samples. Differential gene expression analyses were conducted to extract CDRDEGs, FRDEGs, CRDEGs, AURDEGs, and ARDEGs.

Upon scrutiny of Fig. S10, it is discerned that there exist 78 FRDEGs between male samples and control samples, 66 FRDEGs between female samples and control samples, and 36 FRDEGs between male and female samples. The examination of CRDEGs (Fig. S11) reveals 33 interactions between male samples and control samples, 41 interactions between female samples and control samples, and 14 interactions between male and female samples. Moving on to AURDEGs (Fig. S12), the analysis discloses 55 interactions between male samples and control samples, 40 interactions between female samples and control samples, and 19 interactions between male and female samples. Similarly, ARDEGs (Fig. S13) portray 24 interactions between male samples and control samples, 17 interactions between female samples and control samples, and ten interactions between male and female samples. The exploration of CRDEGs (Fig. S14) manifests 94 interactions between male samples and control samples, 82 interactions between female samples and control samples, and 36 interactions between male and female samples.

In summary, focusing on CDRDEGs, 47 duplicate genes were eliminated in the male sample versus control sample comparison, resulting in a total of 236 CDRDEGs. For female samples versus control, 34 duplicated genes were excluded, leading to the identification of 211 CDRDEGs. The comparative analysis between male and female samples yielded a total of 98 CDRDEGs following the removal of 17 duplicated genes. This delineates a comprehensive overview of the molecular distinctions and shared regulatory elements across the diverse sample cohorts in the context of ankylosing spondylitis.

### Machine learning of CDRDEGs in male and female samples

We computed a total of 236 CDRDEGs differentially expressed between male patient samples and control samples utilizing LASSO, RF, and SVM-RFE machine learning algorithms. The outcomes are presented in Figure S15. LASSO identified 18 characteristic genes (Fig. S15A, B), SVM-RFE identified four characteristic genes (Fig. S15C), and RF identified five characteristic genes (Fig. S15D, E). The intersection of these algorithms yielded three key genes, namely EDEM1, MAP3K11, and TRIM21 (Fig. S15F). Similarly, for female patient samples, 211 CDRDEGs were identified using the same machine learning algorithms, resulting in 22 characteristic genes for LASSO (Fig. S16A, B), 40 for SVM-RFE (Fig. S16C), and five for RF (Fig. S16D, E). The intersection revealed three key genes: COX7B, PEX2, and RHEB (Fig. S16F). Furthermore, 98 CDRDEGs between female and male patient samples were computed, and the intersection of LASSO, RF, and SVM-RFE yielded three key genes: CAPNS1, DDX3X, and TMSB4Y (Fig. S17F).

Upon analyzing the expression of the three key genes between male patient samples and control samples, we observed up-regulation in male patient samples (Fig. S18A–C). Figure S18D illustrates the trend, and Fig. S18E displays high AUC values of 0.895 for EDEM1, 0.925 for MAP3K11, and 0.950 for TRIM21, indicating high accuracy. Subsequently, a comparison of the expression of the three key genes between female patient samples and control samples revealed down-regulation in female patient samples (Fig. S19A–C). Figure S19D depicts the trend, and Fig. S19E displays high AUC values for COX7B (AUC = 0.914), PEX2 (AUC = 0.822), and RHEB (AUC = 0.848), highlighting their high accuracy. Lastly, the expression of the three key genes between male and female patient samples indicated up-regulation of CAPNS1 and TMSB4Y in male samples and down-regulation in female samples, while DDX3X exhibited the opposite pattern (Fig. S20A–C). Figure S20D illustrates the trend, and Fig. S20E shows high AUC values for CAPNS1 (AUC = 0.920) and TMSB4Y (AUC = 0.936), emphasizing their high accuracy.

### The GSEA analysis of key genes and the establishment of a diagnostic efficacy and risk model

Through the application of GSEA analysis, it was discerned that the pivotal gene EDEM1, identified in both male patient samples and control samples, exhibited predominant enrichment in the cell cycle within the high expression group and apoptosis within the low expression group. Notably, MAP3K11 demonstrated notable enrichment in acute myeloid leukemia within the high expression cohort, while associating with Parkinson's disease in the low expression group. The low expression of TRIM21 manifested enrichment in the acute chemokine signaling pathway, whereas high expression TRIM21 was notably associated with Alzheimer's disease (Fig. S21). In the context of female patient samples and control samples, the crucial gene COX7B, when highly expressed, displayed enrichment in Huntington's disease, while its low expression counterpart was enriched in complement and coagulation. PEX2, when highly expressed, exhibited an association with Alzheimer's disease, contrasting with its low expression group, which demonstrated an association with asthma. RHEB, enriched in high expression groups, primarily participated in complement and coagulation cascades, while in low expression groups, it exhibited an association with allograft rejection (Fig. S22). The examination of the gene CAPNS1, in both male and female patient samples, unveiled that high expression levels primarily facilitated antigen processing and presentation. Conversely, in the low expression subset, CAPNS1 predominantly engaged in signaling through B cell receptors. DDX3X, in high expression groups, demonstrated significant enrichment in bladder cancer, particularly in oxidative phosphorylation, whereas its low expression counterpart exhibited this enrichment to a lesser extent. TMSB4Y, in groups with high expression levels, exhibited predominant enrichment in endocytosis, while in low expression groups, it demonstrated predominant enrichment in DNA replication (Fig. S23).

**Figure 7:**
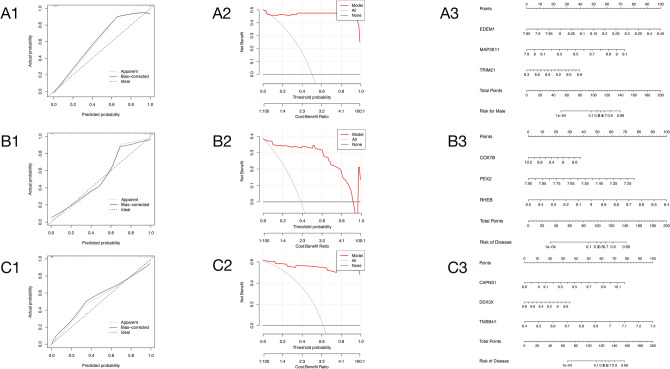
Risk model, calibration curve and decision curve for three key genes in different patient samples and control samples. (**A1**) Calibration curve of EDEM1, MAP3K11 and TRIM21 in male patient samples compared to control samples. (**A2**) Decision curve of EDEM1, MAP3K11 and TRIM21 in male patient samples compared to control samples. (**A3**) Risk model of EDEM1, MAP3K11 and TRIM21 in male patient samples compared to control samples. (**B1**) Calibration curve of COX7B, PEX2 and RHEB in female patient samples compared to control samples. (**B2**) Decision curve of COX7B, PEX2 and RHEB in female patient samples compared to control samples. (**B3**) Risk model of COX7B, PEX2 and RHEB in female patient samples compared to control samples. (**C1**) Calibration curve of CAPNS1, DDX3X and TMSB4Y in male patient samples compared to female patient samples. (**C2**) Decision curve of CAPNS1, DDX3X and TMSB4Y in male patient samples compared to female patient samples. (**C3**) Risk model of CAPNS1,DDX3X and TMSB4Y in male patient samples compared to female patient samples. (**A1–C1**) The horizontal axis of the image represents Predicted probability, the vertical axis represents Actual probability, the short dotted line represents Apparent, and the solid line represents Bias-corrected. The long dotted line represents deal, and the closer the short dotted line and the solid line are to the long dotted line, the higher the credibility of the model is. (**A2–C2**) The horizontal axis represents Threshold probability and Cost : Benefit Ratio, and the vertical axis represents Net Benefit; the red line represents model, the gray line represents all, and the black line represents none. The closer the red line to the gray line, the higher the credibility of the model. (**A3–C3**) The horizontal axis represents the risk of disease, and the vertical axis is the risk of disease caused by each gene and the risk of disease caused by the 3 genes as a whole.

Furthermore, the amalgamation of the three variables into a unified variable facilitated the construction of a risk model by incorporating clinical characteristics (Fig. 7). Figure 7A1–A3 delineates the risk model of key genes in male patient samples vis-à-vis control samples, while Fig. 7B1–B3 illustrates the correlation between crucial genes in female patient samples and control samples. Fig. 7C1–C3 offers a comparative analysis of essential genes within male and female samples. The total score of each gene contributes to the nomogram score, corresponding to distinct AS risks. The adjustment curve (Fig. 7A1, B1, C1) substantiates the accurate predictive ability of nomograms for AS, while the decision curve (Fig. 7A2, B2, C2) analysis reveals the potential of nomograms to provide predictive risk assessment for AS patients. It is evident from the results that the three risk models exhibit a commendable ability to predict the disease.

### Immune cell infiltration and correlation

**Figure 8:**
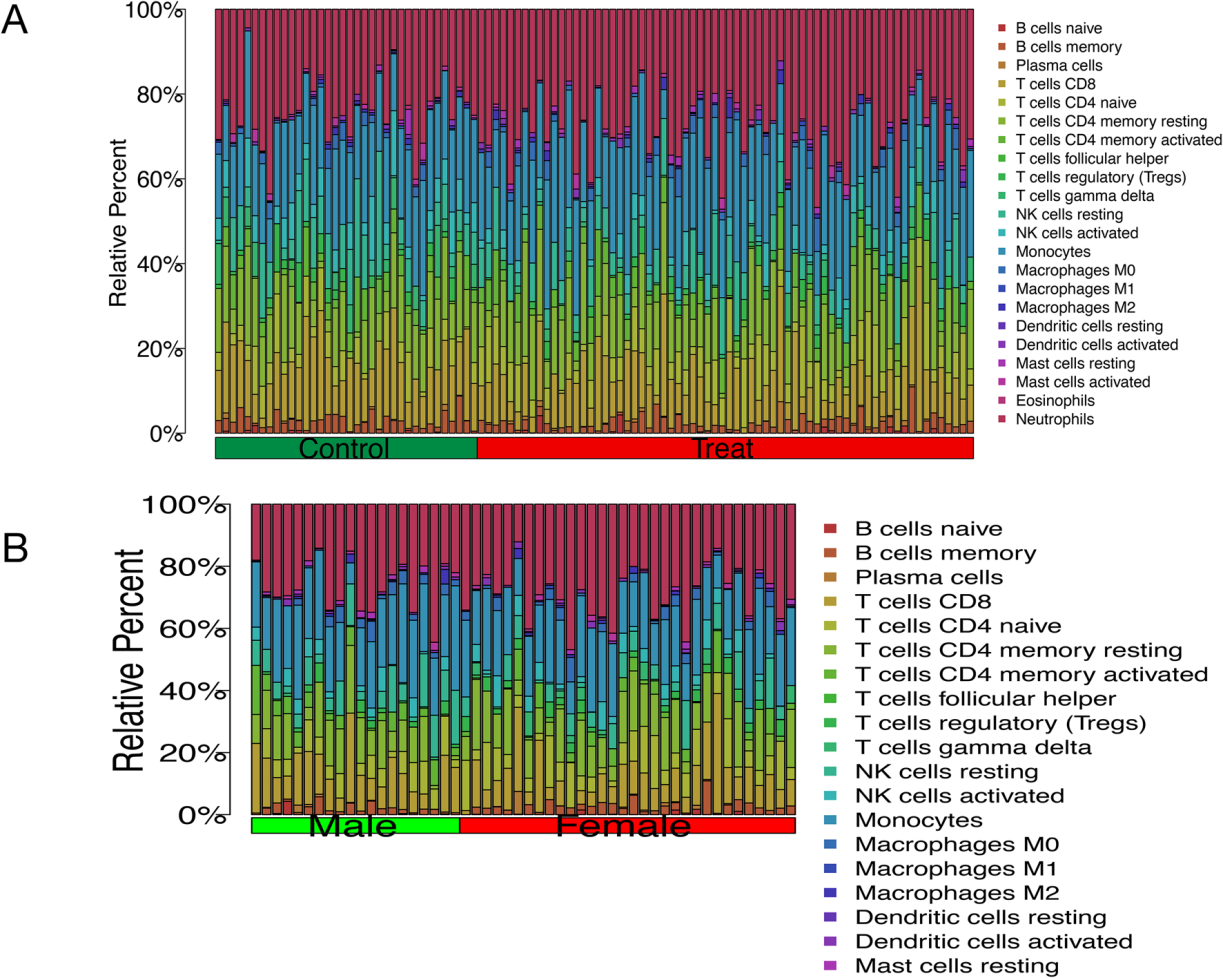
Investigation of immune cells infiltration in patient and control cohort, considering gender diversity. (**A**) Comparison between Ankylosing Spondylitis (AS) patient samples and control samples. (**B**) Stratified analysis based on gender, comparing male samples group and female samples group. (**A**) The horizontal axis of the picture is grouped, the green is the control group, the red is the disease group, and the vertical axis is Relative Percent. The different color squares on the right side represent different immune cells, and the distribution of immune cells in the two groups can be seen in the whole picture. (**B**) The horizontal axis of the picture is grouped, the green is the male group, the red is the female group, and the vertical axis is Relative Percent. The different color squares on the right side represent different immune cells, and the distribution of immune cells in the two groups can be seen in the whole picture.

The outcomes of immune cell infiltration are delineated in Fig. 8, with Fig. 8A presenting the relative proportions of 22 distinct immune cell types in individuals diagnosed with AS in comparison to control samples. Figure 8B further details the immune cell composition in both male and female patients.

The gene EDEM1 demonstrated a substantial inverse association with B cells naive and Neutrophils in terms of immune correlation (R = −0.89, P = 0.012 with neutrophils; R = −0.86, P = 0.024 with B cells naive). This observation underscores its pivotal role in distinguishing male patient samples from control samples. Moreover, a noteworthy correlation was identified between MAP3K11 and T cells CD4 memory activated, as well as plasma cells. The correlation coefficient (R) for MAP3K11 and plasma cells was −0.89, with a p-value of 0.0073 (Fig. S24A1–A3). Furthermore, the correlation coefficient (R) for MAP3K11 and T cells CD4 memory activated was −0.76, with a p-value of 0.049 (Fig. S24B1–B3). TRIM21 manifested a significant positive correlation with both resting and activated mast cells, while exhibiting a significant negative correlation with T cells follicular helper. The correlation coefficients (R) were determined as 0.89 (P = 0.012) with mast cells activated, 1 (P = 4e−04) with mast cells resting, and −0.8 (P = 0.03) with T cells follicular helper (Fig. S24C1–C4).

The findings of this investigation reveal a noteworthy positive correlation (R = 0.36, P = 0.043) between COX7B and Eosinophils in both female patient samples and control samples, indicating an immune correlation (Fig. S24D1–D2). Additionally, a statistically significant positive correlation was noted between PEX2 and Monocytes, as well as Macrophages M0 (R = 0.36, R = 0.046 with Monocytes, R = 0.35, P = 0.047 with Macrophages M0) (Fig. S24E1–E3). RHEB exhibited significant positive correlations with T cells regulatory (Tregs) and B cells memory, and significant negative correlations with Macrophages M1, NK cells resting, and T cells CD4. Notably, a significant negative correlation was observed between B cells memory, Macrophages M1, and NK cells resting with RHEB. Conversely, a significant positive correlation was found between T cells CD4 memory activated and RHEB (R = 0.52, P = 0.0021 with T cells regulatory (Tregs), and R = −0.49 with Macrophages M1. P = 0.0044, R = −0.44, P = 0.012 with NK cells resting, R = −0.39, P = 0.027 with T cells CD4 memory activated) (Fig. S24F1–F6).

CAPNS1, the key gene between male and female patient samples, and in terms of immune correlation, it was significantly positively correlated with NK cells resting and T cells CD4 memory activated, and significantly negatively correlated with Neutrophils and T cells CD4 naive, with NK cells resting R = 0.42, P = 0.002, and T cells CD4 memory activated R = 0.32. P = 0.021 (Fig. S24G1–G5). In relation to immune correlation, DDX3X exhibited a positive correlation with neutrophils and macrophages M0, while displaying a negative correlation with T cells CD8, R = 0.31, P = 0.027 with Neutrophils, R = 0.29, P = 0.038 with Macrophages M0. DDX3X was negatively correlated with T cells CD8, R = 0.31, P = 0.027 with Neutrophils, R = 0.29, P = 0.038 with Macrophages M0. R = −0.35, P = 0.011 (Fig. S24H1–H4). There was a significant positive correlation observed between the levels of resting NK cells and T cells gamma delta with the expression of TMSB4Y (R = 0.39, P = 0.0038) (Fig. S24I1–I3).

Table 2 shows the expression of five CDRDEGs in the control group of the disease group and the female group of the male group. ↑ represents high gene expression and ↓ represents low gene expression.

**Table 2:** The three groups of samples were compared with the final CDRDEGs results.

	AS and healthy control group		Male patient samples and healthy controls		Female patient samples and healthy controls		Male patient samples and female patient samples	
	AS	Healthy control	Male patient	Healthy control	Female patient	Healthy control	Male patient	Female patient
FRDEGs	CLIC4↑	CLIC4↓	MAP3K11↑	MAP3K11↓	PEX2↓	PEX2↑	TMSB4Y↑	TMSB4Y↓
			TRIM21↑	TRIM21↓				
CRDEGs	SLC25A5↓	SLC25A5↑			COX7B↓	COX7B↑		
AURDEGs	EDEM1↑	EDEM1↓	EDEM1↑	EDEM1↓	RHEB↓	RHEB↑	CAPNS1↑	CAPNS1↓
ARDEGs								
PRDEGs	BIRC2↑	BIRC2↓	TRIM21↑	↓			DDX3X↓	DDX3X↑
	MATK↓	MATK↑						
	PKN2↑	PKN2↓						

## Discussion

Ankylosing spondylitis (AS) represents a chronic inflammatory ailment of elusive origin. Recent investigations, as referenced by^29^, have furnished compelling evidence bolstering the assertion of an autoimmune mechanism's involvement in the pathogenesis of this condition. Within the domains of epidemiology, clinical symptomatology, immune response, hormonal levels, diagnostic frequencies, therapeutic outcomes, and prognostic indicators, discernible nuances emerge when comparing male and female cohorts sharing the AS condition^30–32^. The male-to-female patient ratio approximates 5:1. Moreover, female patients tend to manifest a more gradual onset of symptoms, chiefly characterized by alterations in disease activity and clinical manifestations^33^. Conversely, male patients exhibit a swifter disease progression^34^. It is crucial to emphasize that notwithstanding the more gradual progression, female patients may endure a heightened overall disease burden and greater functional impairment^35^. In the context of treatment and prognosis, female patients demonstrate a less favorable clinical response to TNF inhibitor therapy and diminished drug retention compared to their male counterparts^36,37^. Consequently, a proactive approach to addressing the treatment requirements of female AS patients and intensifying diagnostic scrutiny is imperative to preclude delays in diagnosis. In light of these considerations, the current investigation embarked on a comprehensive bioinformatics exploration to scrutinize the DEGs and pathways associated with immune cell infiltration during the evolution and progression of AS across distinct genders. This underscores the necessity of factoring in gender differences in the diagnosis and treatment of AS, advocating for a personalized treatment approach tailored to male and female patients.

Unraveling the fundamental determinants contributing to this gender asymmetry is pivotal for adept identification and management of the malady. In view of the aforementioned dissimilarities, delving into potential therapeutic targets for AS through a gender-specific prism holds considerable promise in the realm of research. By meticulously considering the distinct factors influencing the disease in males and females, the prospect of pinpointing specific targets and devising more customized treatment modalities becomes conceivable. In essence, the presence of autoantibodies and the observed gender differentials in AS illuminate potential avenues for research and therapeutic interventions, underscoring the importance of scrutinizing the disease through a gender-specific lens. Both inflammation and immune cells assume pivotal roles in ankylosing spondylitis. The immune system and inflammatory cells stand as integral elements in the pathogenesis of ankylosing spondylitis^38,39^. The comprehensive outcomes of all CDRDEGs are delineated in Table 2.

Potential advancements in the diagnosis and treatment of AS may emerge through the identification of immunoinflammatory and cell death-related biomarkers. Employing a heatmap, differential violin map, and gene correlation map, we conducted an exhaustive analysis to pinpoint differential genes associated with the seedless cell death pattern. Subsequently, 216 CDRDEGs were deduced after eliminating duplicate genes, and the samples underwent clustering using a consistent clustering method. The relative changes in consensus matrix diagrams, CDF diagrams, and area and trajectory diagrams under CDF curves were categorized into two distinct groups. Utilizing four machine learning algorithms, we selected the optimal score-fitting model and, in conjunction with the LASSO algorithm, identified 6 CDRDEGs linked to AS disease: CLIC4, BIRC2, MATK, PKN2, SLC25A5, and EDEM1. Among these, CLIC4 belongs to FRDEGs, EDEM1 to AURDEGs, and BIRC2, MATK, and PKN2 to PRDEGs; MATK and SLC25A5 are down-regulated genes, while the rest are up-regulated. BIRC2 aligns with prior research findings^40^. Upon further analysis of these six pivotal disease genes, it was observed that the biological processes of natural killer cell-mediated immunity and glycosyl compound biosynthesis were significantly up-regulated, while the molecular function of transcriptional coactivator binding was down-regulated. Furthermore, spliceosomes of the TCA cycle, and three pathways related to a carbon pool in the folic acid and citric acid cycle were significantly up-regulated, whereas the mTOR signaling pathway, glyceride metabolism, and gnrh signaling pathway were significantly down-regulated. BIRC2 and EDEM1 exhibit co-expression and have been identified as direct targets of miR-204^41^, consistent with our research findings.

Elevated expression of EDEM1, MAP3K11, and TRIM21 was observed in male patient samples, with TRIM21 belonging to both FRDEGs and PRDEGs. Conversely, COX7B, PEX2, and RHEB were down-regulated genes in female patient samples, while CAPNS1 and TMSB4Y were up-regulated in male patient samples, and DDX3X exhibited down-regulation in male patient samples and up-regulation in female patient samples. Research indicates that miRNA-199a-5p directly targets RHEB, inhibiting RHEB and subsequently decreasing mTOR phosphorylation and inducing autophagy. The overexpression of miRNA-199a-5p inhibits mTOR signaling, a effect mitigated by pcDNA3.1-RHEB introduction. Additionally, patients with advanced spinal damage in AS showed reduced autophagy levels, suggesting that miRNA-199a-5p may modulate mTOR signaling, induce autophagy, and inhibit AS pathogenesis by directly targeting RHEB^42^. Studies have identified DDX3Y and TMSB4Y as common genes in the dorsolateral prefrontal cortex of patients with Alzheimer's disease (AD) or major depressive disorder (MDD)^43^. Investigating potential gender differences in the risk of AD and MDD among male and female AS patients warrants further exploration.

Immune cell infiltration exhibited heightened activity in female AS patients compared to their male counterparts, as indicated by the results of immune cell infiltration analysis. Female patients displayed an elevated proportion of immune cells in comparison to normal healthy controls, prompting exploration into whether this suggests a lower prevalence among females^44^. Notably, the immune correlation analysis revealed a significant negative correlation between EDEM1 and B cells naive, as well as Neutrophils. Additionally, MAP3K11 demonstrated a notable negative correlation with T cells CD4 memory activated and Plasma cells, while exhibiting a positive correlation with both resting and activated mast cells, and a negative correlation with follicular helper T cells TRIM21. Furthermore, COX7B exhibited a significant positive correlation with eosinophils in terms of immune correlation. Immune correlation analyses also unveiled a positive correlation between Macrophages, monovates, and PEX2. Conversely, RHEB demonstrated a positive correlation with T cells regulatory (Tregs) and B cells memory, but a negative correlation with Macrophages M1, NK cells resting, and T cells CD4 memory activated. CAPNS1 displayed significant positive correlations with resting NK cells and CD4 memory-activated T cells, while exhibiting negative correlations with neutrophils and CD4 naive T cells. Moreover, DDX3X displayed a negative correlation with T cells CD8. Interestingly, TMSB4Y demonstrated positive correlations with NK cells resting and T cells gamma delta. In summary, a noteworthy observation was the negative correlation of key genes in male and female patient samples with T cells CD4 memory activated. Intriguingly, the key genes exhibited a positive correlation with T cells CD4 memory activated when comparing male and female patient samples, and the correlation with NK cells resting showed an opposite and robust pattern, warranting further investigation.

In the investigation of sex-specific biomarkers pertaining to programmed cell death and the analysis of immune cells in ankylosing spondylitis (AS), various pertinent factors have been unveiled. It is imperative to underscore that the prevalence of AS is notably higher in males as opposed to females, and discernible sex-related variations in immune responses may contribute to this discernment. Observations from studies have detected anomalies in apoptosis-related proteins in AS, with identified potential biomarkers encompassing caspases (specifically, caspase-3 and caspase-8) and death receptors (notably, Fas). Both adaptive and innate immune cells intricately partake in the context of AS, with T cells (comprising CD4+ and CD8+ T cells), B cells, natural killer (NK) cells, and dendritic cells being discerned as subjects of alteration through immune profiling endeavors. While distinctions in immune cell subsets between genders may be present, further exploration is requisite to validate such disparities. AS manifests sex-specific nuances in disease manifestation, gravity, and responsiveness to treatment. Hormonal, genetic, and environmental elements collectively contribute to these divergences. Ongoing research delves into elucidating the impact of sex hormones, such as estrogen and testosterone, on the modulation of immune responses and programmed cell death. State-of-the-art methodologies, including genomics, transcriptomics, proteomics, and metabolomics, are instrumental in the identification of potential sex-specific biomarkers and the unraveling of immune cell dysregulation in the context of AS. The molecular intricacies underpinning the pathogenesis of the disease stand poised for elucidation through the application of these advanced approaches.

## Conclusion

In essence, an exhaustive bioinformatics investigation was undertaken to scrutinize DEGs and pathways linked to immune cell infiltration in the initiation and progression of AS. Within this inquiry, we executed the identification and scrutiny of pivotal regulatory genes and pathways implicated in cellular apoptosis. The revelations derived from this inquiry bear consequential ramifications for elucidating the intricate molecular pathways underpinning AS and pinpointing potential molecular targets for therapeutic intervention. By delving into the molecular intricacies and pathways associated with AS, the prospect of enhancing early detection and diagnostic precision of the ailment comes to the fore. Furthermore, this examination possesses the potential to introduce groundbreaking perspectives concerning the prevalence and progression of AS within diverse genders, accentuating the pivotal nature of incorporating gender-specific factors into the comprehensive understanding of AS. In the larger context, the bioinformatics exploration into DEGs and pathways pertinent to AS has substantially enriched our comprehension of the malady, thereby laying the foundation for subsequent investigations and plausible therapeutic modalities. It is noteworthy that ongoing research into sex-specific biomarkers and immune cell analysis in AS remains a dynamic area of inquiry, with the possibility of novel findings emerging as studies continue to advance our collective understanding of this intricate ailment.

### Advantages and limitations

To the best of our current understanding, this investigation represents the inaugural exploration into gender-specific biomarkers associated with programmed cell death in ankylosing spondylitis. The primary aim is to delineate distinctions in programmed cell death-related genes between male and female patients, concurrently delving into their immune cell analysis. Nevertheless, multiple constraints impede the research outcomes, encompassing an insufficient sample size and the considerable confounding influence of external variables, thereby introducing complexity to the interpretation of the findings. The molecular intricacies underpinning the identified biomarkers remain partially elucidated, necessitating further experimental scrutiny. Furthermore, given the absence of clinical patients in this study, the diagnostic potential of the identified genes for AS across different genders was indirectly assessed. Consequently, additional prospective investigations are imperative to corroborate these discoveries and facilitate their translation into clinical applications.

### Supplementary Information

Supplementary Figures.

## Data Availability

Datasets accessible to the public were scrutinized in the course of this investigation, and they can be accessed at the following identifiers: GSE25101 and GSE73754 (https://www.ncbi.nlm.nih.gov/geo/). For those interested, the data generated in the context of this study can be obtained upon request from the corresponding author.

## References

[1] Sieper J, Poddubnyy D (2017). Axial spondyloarthritis. Lancet.

[2] McVeigh CM, Cairns AP (2006). Diagnosis and management of ankylosing spondylitis. BMJ.

[3] Woodward LJ, Kam PC (2009). Ankylosing spondylitis: Recent developments and anaesthetic implications. Anaesthesia.

[4] Ensslin C, Micheroli R, Kissling S, Götschi A, Bürki K, Bräm R, de Hooge M, Baraliakos X, Nissen MJ, Möller B, Exer P, Andor M, Distler O, Scherer A, Ciurea A (2023). Impact of sex on spinal radiographic progression in axial spondyloarthritis: A longitudinal Swiss cohort analysis over a period of 10 years. RMD Open.

[5] Wang C, He Y, Zheng J, Wang X, Chen S (2023). Dissecting order amidst chaos of programmed cell deaths: Construction of a diagnostic model for KIRC using transcriptomic information in blood-derived exosomes and single-cell multi-omics data in tumor microenvironment. Front. Immunol..

[6] Chen W, Chen Y, Wu L, Gao Y, Zhu H, Li Y, Ji X, Wang Z, Wang W, Han L, Zhu B, Wang H, Xu M (2023). Identification of cell death-related biomarkers and immune infiltration in ischemic stroke between male and female patients. Front. Immunol..

[7] Lee SH, Lee YA, Woo DH, Song R, Park EK, Ryu MH, Kim YH, Kim KS, Hong SJ, Yoo MC, Yang HI (2006). Association of the programmed cell death 1 (PDCD1) gene polymorphism with ankylosing spondylitis in the Korean population. Arthritis Res. Ther..

[8] Zheng J, Conrad M (2020). The metabolic underpinnings of ferroptosis. Cell Metab..

[9] Li Q, Chen Z, Yang C, Wang L, Ma J, He T, Li H, Quan Z (2022). Role of ferroptosis-associated genes in ankylosing spondylitis and immune cell infiltration. Front. Genet..

[10] Chang S, Tang M, Zhang B, Xiang D, Li F (2022). Ferroptosis in inflammatory arthritis: A promising future. Front. Immunol..

[11] Taddei ML, Giannoni E, Fiaschi T, Chiarugi P (2012). Anoikis: An emerging hallmark in health and diseases. J. Pathol..

[12] Kucuksezer UC, Aktas Cetin E, Esen F, Tahrali I, Akdeniz N, Gelmez MY, Deniz G (2021). The role of natural killer cells in autoimmune diseases. Front. Immunol..

[13] Jayson MI, Davis P, Whicher JT, Walters G (1975). Serum copper and caeruloplasmin in ankylosing spondylitis, systemic sclerosis, and morphea. Ann. Rheum. Dis..

[14] Aiginger P, Kolarz G, Willvonseder R (1978). Copper in ankylosing spondylitis and rheumatoid arthritis. Scand. J. Rheumatol..

[15] Aiginger P, Kolarz G, Willvonseder R (1978). Copper in ankylosing spondylitis and rheumatoid arthritis. Scand. J. Rheumatol..

[16] Percival SS (1998). Copper and immunity. Am. J. Clin. Nutr..

[17] Zhao YG, Codogno P, Zhang H (2021). Machinery, regulation and pathophysiological implications of autophagosome maturation. Nat. Rev. Mol. Cell Biol..

[18] Zeng C, Wang S, Chen F, Wang Z, Li J, Xie Z, Ma M, Wang P, Shen H, Wu Y (2023). Alpinetin alleviates osteoporosis by promoting osteogenic differentiation in BMSCs by triggering autophagy via PKA/mTOR/ULK1 signaling. Phytother. Res..

[19] Chen Y, Wu Y, Fang L, Zhao H, Xu S, Shuai Z, Yu H, Cai G, Zhan HQ, Pan F (2023). METTL14-m6A-FOXO3a axis regulates autophagy and inflammation in ankylosing spondylitis. Clin. Immunol..

[20] Tan M, Zhang QB, Liu TH, Yang YY, Zheng JX, Zhou WJ, Xiong Q, Qing YF (2020). Autophagy dysfunction may be involved in the pathogenesis of ankylosing spondylitis. Exp Ther Med..

[21] Ma C, Wen B, Zhang Q, Shao PP, Gu W, Qu K, Shi Y, Wang B (2019). Emodin induces apoptosis and autophagy of fibroblasts obtained from patient with ankylosing spondylitis. Drug Des. Dev. Ther..

[22] D'Arcy MS (2019). Cell death: A review of the major forms of apoptosis, necrosis and autophagy. Cell Biol. Int..

[23] Li X, Li X, Wang H, Zhao X (2023). Exploring hub pyroptosis-related genes, molecular subtypes, and potential drugs in ankylosing spondylitis by comprehensive bioinformatics analysis and molecular docking. BMC Musculoskelet. Disord..

[24] Thakur AK, Luthra-Guptasarma M (2022). Differences in cellular clearing mechanisms of aggregates of two subtypes of HLA-B27. Front. Immunol..

[25] Matsuzawa-Ishimoto Y, Hwang S, Cadwell K (2018). Autophagy and inflammation. Annu. Rev. Immunol..

[26] Kanehisa M, Goto S (2000). KEGG: Kyoto encyclopedia of genes and genomes. Nucleic Acids Res..

[27] Kanehisa M (2019). Toward understanding the origin and evolution of cellular organisms. Protein Sci..

[28] Kanehisa M, Furumichi M, Sato Y, Kawashima M, Ishiguro-Watanabe M (2023). KEGG for taxonomy-based analysis of pathways and genomes. Nucleic Acids Res..

[29] Mauro D, Thomas R, Guggino G, Lories R, Brown MA, Ciccia F (2021). Ankylosing spondylitis: An autoimmune or autoinflammatory disease?. Nat. Rev. Rheumatol..

[30] Unal Enginar A (2023). A comparison of the clinical characteristics and quality of life of male and female patients with non-radiographic axial spondyloarthritis. Int. Immunopharmacol..

[31] Hochberg MC (2022). Seminars in arthritis and rheumatism moves to online-only publication. Semin. Arthritis Rheum..

[32] Gracey E, Yao Y, Green B, Qaiyum Z, Baglaenko Y, Lin A, Anton A, Ayearst R, Yip P, Inman RD (2016). Sexual dimorphism in the Th17 signature of ankylosing spondylitis. Arthritis Rheumatol..

[33] Walsh JA, Magrey M (2021). Clinical manifestations and diagnosis of axial spondyloarthritis. J. Clin. Rheumatol..

[34] Fish EN (2008). The X-files in immunity: Sex-based differences predispose immune responses. Nat. Rev. Immunol..

[35] Purnamawati K, Ong JA, Deshpande S, Tan WK, Masurkar N, Low JK, Drum CL (2018). The importance of sex stratification in autoimmune disease biomarker research: A systematic review. Front. Immunol..

[36] Tournadre A, Pereira B, Lhoste A, Dubost JJ, Ristori JM, Claudepierre P, Dougados M, Soubrier M (2013). Differences between women and men with recent-onset axial spondyloarthritis: Results from a prospective multicenter French cohort. Arthritis Care Res. (Hoboken)..

[37] Ogdie A, Benjamin Nowell W, Reynolds R, Gavigan K, Venkatachalam S, de la Cruz M, Flood E, Schwartz EJ, Romero B, Park Y (2019). Real-world patient experience on the path to diagnosis of ankylosing spondylitis. Rheumatol. Ther..

[38] Tavasolian F, Lively S, Pastrello C, Tang M, Lim M, Pacheco A, Qaiyum Z, Yau E, Baskurt Z, Jurisica I, Kapoor M, Inman RD (2023). Proteomic and genomic profiling of plasma exosomes from patients with ankylosing spondylitis. Ann. Rheum. Dis..

[39] Wang H, Luo F, Shao X, Gao Y, Jiang N, Jia C, Li H, Chen R (2023). Integrated proteomics and single-cell mass cytometry analysis dissects the immune landscape of ankylosing spondylitis. Anal. Chem..

[40] Xu ZY, Zhou C, Zhang KF, Zheng YP (2018). Identification of key genes in ankylosing spondylitis. Immunol. Lett..

[41] Li G, Luna C, Qiu J, Epstein DL, Gonzalez P (2011). Role of miR-204 in the regulation of apoptosis, endoplasmic reticulum stress response, and inflammation in human trabecular meshwork cells. Invest. Ophthalmol. Vis. Sci..

[42] Wang Y, Luo J, Wang X, Yang B, Cui L (2017). MicroRNA-199a-5p induced autophagy and inhibits the pathogenesis of ankylosing spondylitis by modulating the mTOR signaling via directly targeting Ras homolog enriched in brain (Rheb). Cell Physiol. Biochem..

[43] Rastad S, Barjaste N, Lanjanian H, Moeini A, Kiani F, Masoudi-Nejad A (2023). Parallel molecular alteration between Alzheimer's disease and major depressive disorder in the human brain dorsolateral prefrontal cortex: An insight from gene expression and methylation profile analyses. Genes Genet. Syst..

[44] Sun X, Zhou C, Chen L, Huang S, Ye Z, Yi M, Liao S, Li H, Jiang J, Chen J, Chen W, Chen T, Guo H, Zhang S, Zhu J, Liang T, Zhan X, Liu C (2023). Epidemiological characteristics of ankylosing spondylitis in Guangxi Province of China from 2014 to 2021. Arch. Med. Sci..

