# Supplementary material for: Identification of sex-specific biomarkers related to programmed cell death and analysis of immune cells in ankylosing spondylitis
Source: Sci Rep. 2024 Jul 4;14:15358. doi: 10.1038/s41598-024-65745-3 (PMC11224221; doi:10.1038/s41598-024-65745-3)

supplementary data

Identification of sex-specific biomarkers related to programmed cell death and analysis of immune cells in ankylosing spondylitis

The original HD versions of all figures are in the supplementary data pack

FIG.S1 Identification of biomarkers of cuproptosis-related DEGs (CRDEGs) between disease and healthy samples

FIG.S1

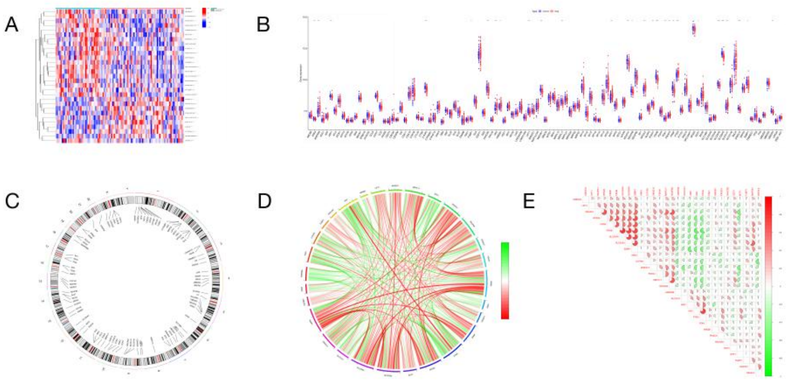

A: Heat map comparing the disease and healthy samples.

B: Violin plots illustrating the contrast between the disease and healthy samples.

C: Chromosomal loci distribution of CRDEGs in the disease and healthy samples.

D: Association map of CRDEGs between the disease and healthy groups.

E: Correlation map depicting the relationship among CRDEGs in the disease and healthy groups.

FIG.S2 Identification of biomarkers of autophagy-related DEGs (AURGs) between disease and healthy samples

FIG.S2

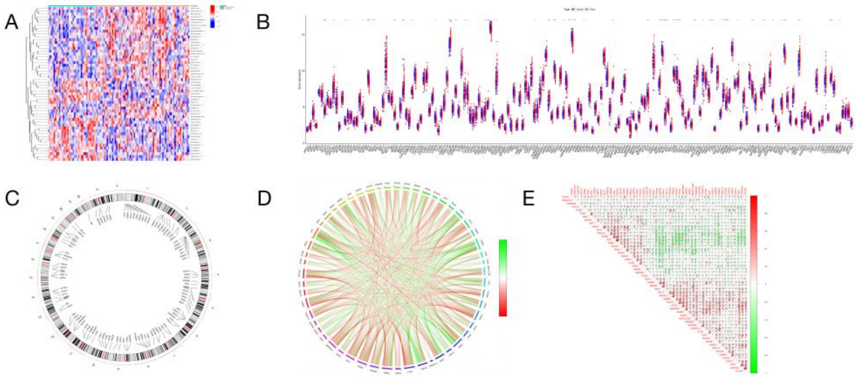

A: Heat map comparing the disease and healthy samples.

B: Violin plots illustrating the contrast between the disease and healthy samples.

C: Chromosomal loci distribution of AURGs in the disease and healthy samples.

D: Association map of AURGs between the disease and healthy groups.

E: Correlation map depicting the relationship among AURGs in the disease and healthy groups.

FIG.S3 Identification of biomarkers of anoikis-related DEGs (ARDEGs) between disease and healthy samples

FIG.S3

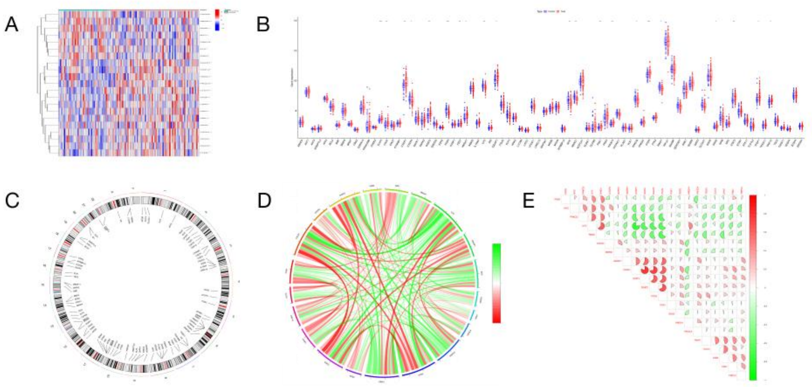

A: Heat map comparing the disease and healthy samples.

B: Violin plots illustrating the contrast between the disease and healthy samples.

C: Chromosomal loci distribution of ARDEGs in the disease and healthy samples.

D: Association map of ARDEGs between the disease and healthy groups.

E: Correlation map depicting the relationship among ARDEGs in the disease and healthy groups.

FIG.S4 Identification of biomarkers of pyroptosis-related DEGs (PRDEGs) between disease and healthy samples

FIG.S4

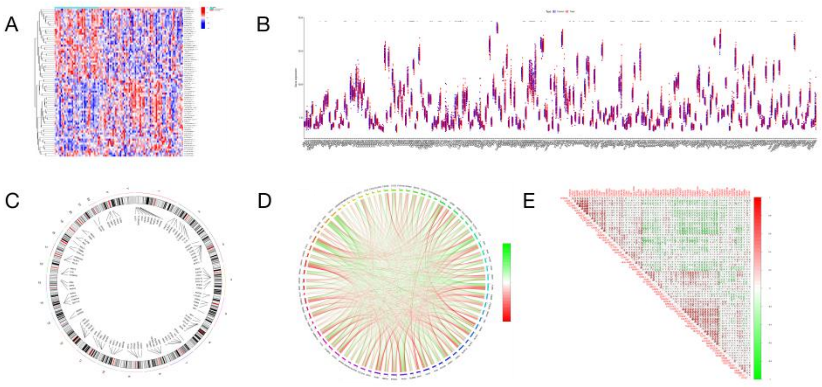

A: Heat map comparing the disease and healthy samples.
B: Violin plots illustrating the contrast between the disease and healthy samples.
C: Chromosomal loci distribution of PRDEGs in the disease and healthy samples.
D: Association map of PRDEGs between the disease and healthy groups.
E: Correlation map depicting the relationship among PRDEGs in the disease and healthy groups.

FIG.S5 Cluster analysis and machine learning analysis graph between disease group and healthy control group

FIG.S5

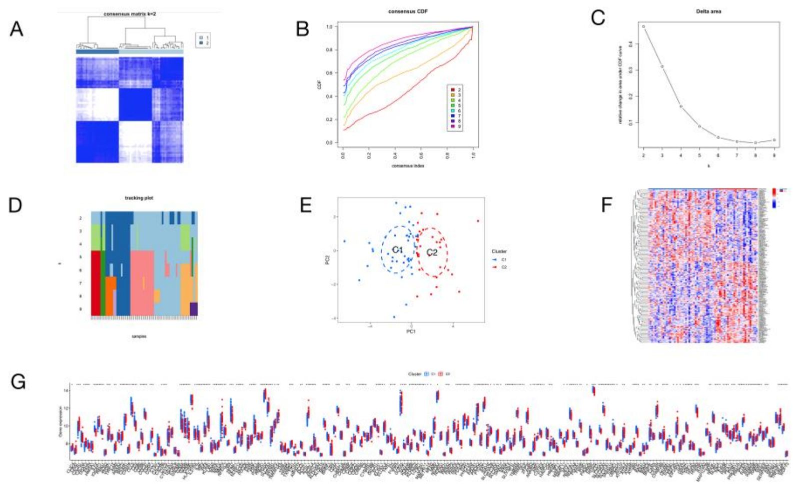

A: Display the heat map of the agreement matrix for k = 2.

B:Consensus CDF at k = 2 – 9.

C:Relative change in area under the CDF curve.

D:Trace plot of sample classification when k = 2 – 9.

E:The PCA plot demonstrates that CDRDEGs successfully categorize patients with AS into two subgroups (C1 and C2).

F:The heat map of consistent clustering

G:Violin diagram of consistent clustering

FIG.S6 Four different types of machine learning were utilized to select key biomarkers associated with cellular demise.

FIG.S6

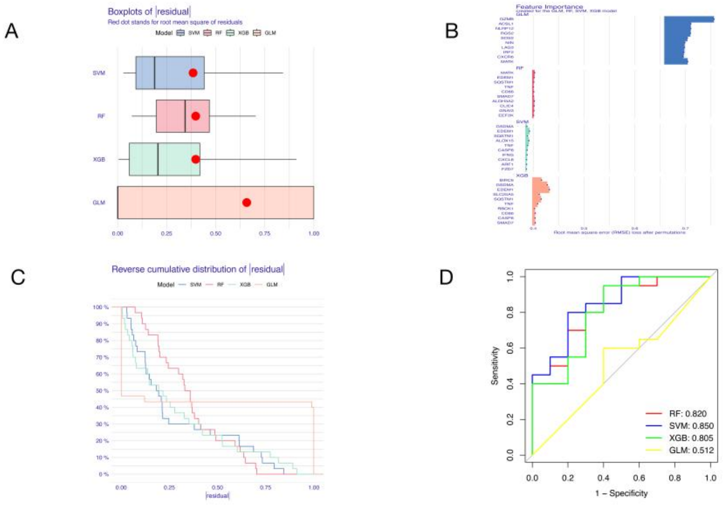

A : The residual box plot of the four models.

B : The importance of features created by the four models.

C : The cumulative distribution of difference and inverse of the four models.

D: ROC of the four models.

FIG.S7 Three types of machine learning were used to select core biomarkers associated with cellular demise.

FIG.S7

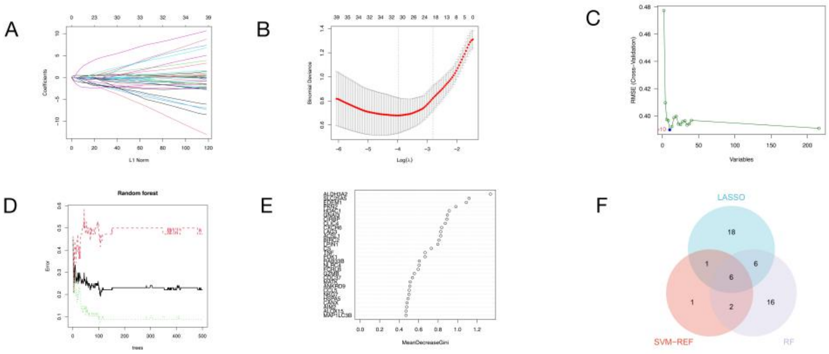

A:Ten iterations of cross validation were conducted to optimize parameter selection in the LASSO model. Each curve represents a specific gene.

B:Analysis of LASSO coefficients is conducted, with a vertical dotted line indicating the optimal lambda.

C:The correlation between the quantity of random forest trees and the rate of error.

D:Assessing the significance of different genes.

E:The SVM-RFE algorithm is used for selecting feature genes.

F:The Venn diagram illustrates the common genes among LASSO, random forest, and SVM-RFE algorithm.

FIG.S8 The variations in expression of six crucial genes were compared between the disease group and the healthy group, along with their respective levels of sensitivity and specificity.(BIRC2 ,EDEM1 and CLIC4)

FIG.S8

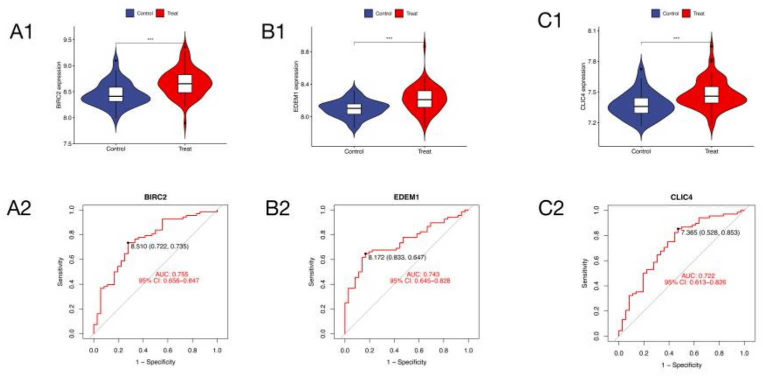

A1 : Expression of BIRC2 between the disease and healthy groups. BIRC2 was up-regulated in the disease.

A2 : ROC curve of BIRC2 gene, AUC=0.755.

B1 : Expression of EDEM1 between the disease and healthy groups. EDEM1 was up-regulated in the disease.

B2 : ROC curve of EDEM1 gene, AUC = 0.743.

C1 : Expression of CLIC4 between the disease and healthy groups. CLIC4 was up-regulated in the disease,.

C2 : ROC curve of CLIC4 gene,AUC =0.722.

FIG.S9 GSEA analysis of six key genes(EDEM1 ,CLIC4 and BIRC2)

FIG.S9

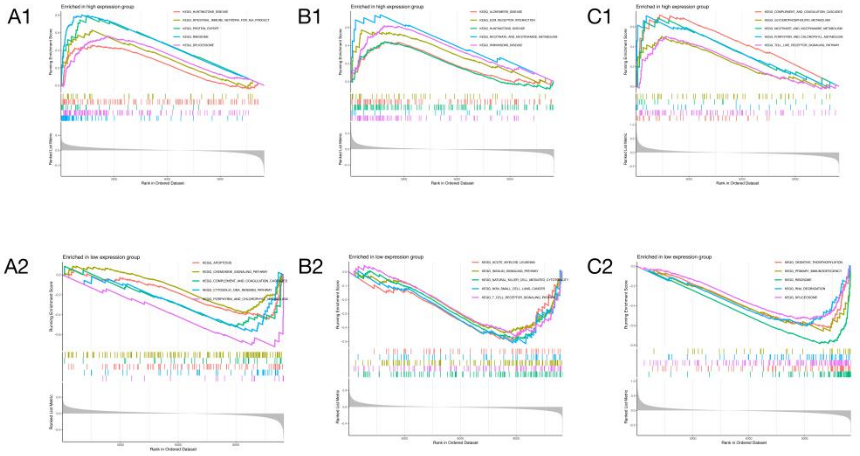

A1 : GSEA analysis of EDEM1 in high expression group
A2 : GSEA analysis of EDEM1 in low expression group
B1 : GSEA analysis of CLIC4 in high expression group
B2 : GSEA analysis of CLIC4 in low expression group
C1 : GSEA analysis of BIRC2 in high expression group
C2 : GSEA analysis of BIRC2 in low expression group

FIG.S10 A1-A3 The heat map of male and female ferroptosis-related DEGs(FRDEGs)

FIG.S10

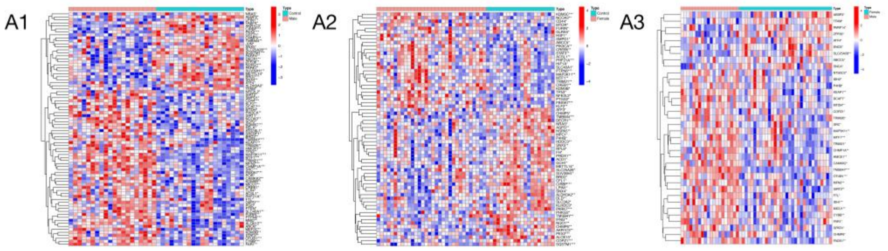

A1: The heat map of FRDEGs between male samples and control samples.
A2: The heat map of FRDEGs between female samples and control samples.
A3: The heat map of FRDEGs between male samples and female samples.

FIG.S10 A4-A6 Violin plot of male and female ferroptosis-related DEGs(FRDEGs)

FIG.S10

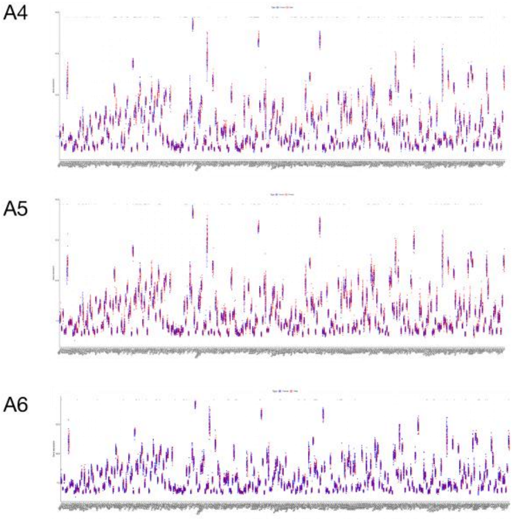

A4: Violin plot of FRDEGs between male samples and control samples.
A5: Violin plot of FRDEGs between female samples and control samples.
A6: Violin plot of FRDEGs between male samples and female samples.

FIG.S11 A1-A3 The heat map of male and female cuproptosis-related DEGs(CRDEGs)

FIG.S11

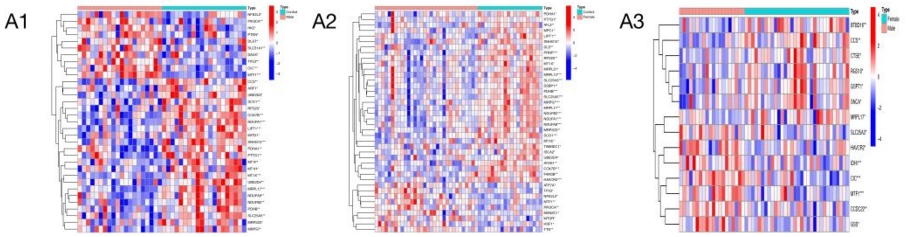

A1: The heat map of CRDEGs between male samples and control samples.
A2: The heat map of CRDEGs between female samples and control samples.
A3: The heat map of CRDEGs between male samples and female samples.

FIG.S11 A4-A6 Violin plot of male and female cuproptosis-related DEGs(CRDEGs)

FIG.S11

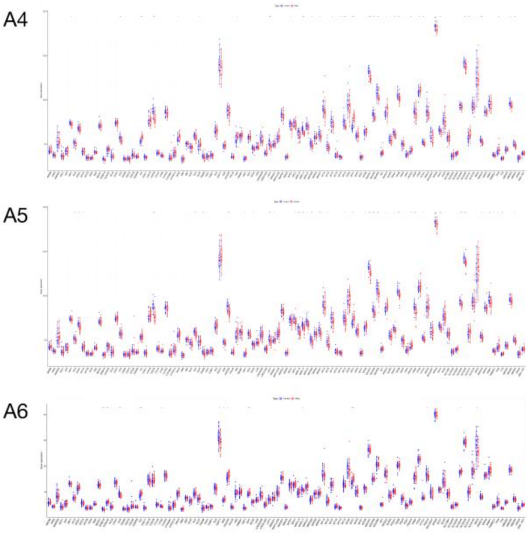

A4: Violin plot of CRDEGs between male samples and control samples.
A5: Violin plot of CRDEGs between female samples and control samples.
A6: Violin plot of CRDEGs between male samples and female samples.

FIG.S12 A1-A3 The heat map of male and female autophagy-related DEGs (AURDEGs)

FIG.S12

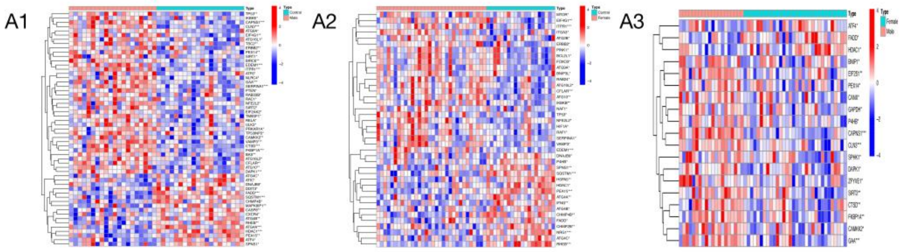

A1: The heat map of AURDEGs between male samples and control samples.
A2: The heat map of AURDEGs between female samples and control samples.
A3: The heat map of AURDEGs between male samples and female samples.

FIG.S12 A4-A6 Violin plot of male and female autophagy-related DEGs (AURDEGs)

FIG.S12

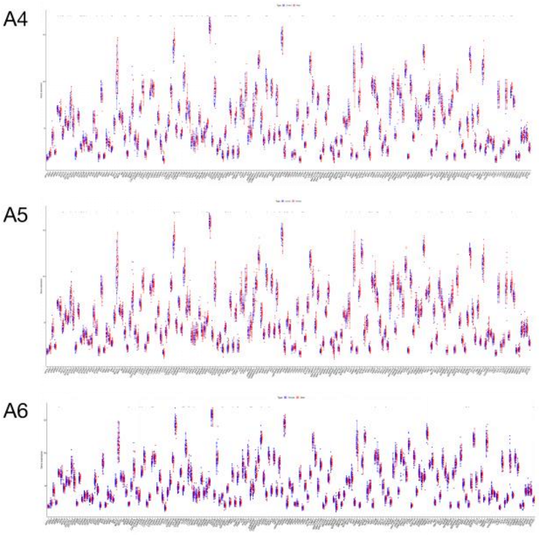

A4: Violin plot of AURDEGs between male samples and control samples.
A5: Violin plot of AURDEGs between female samples and control samples.
A6: Violin plot of AURDEGs between male samples and female samples.

FIG.S13 A1-A3 The heat map of male and female anoikis-related DEGs (ARDEGs)

FIG.S13

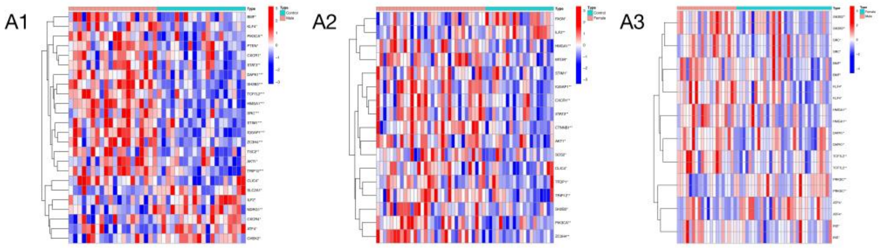

A1: The heat map of ARDEGs between male samples and control samples.
A2: The heat map of ARDEGs between female samples and control samples.
A3: The heat map of ARDEGs between male samples and female samples.

FIG.S13 A4-A6 Violin plot of male and female anoikis-related DEGs (ARDEGs)

FIG.S13

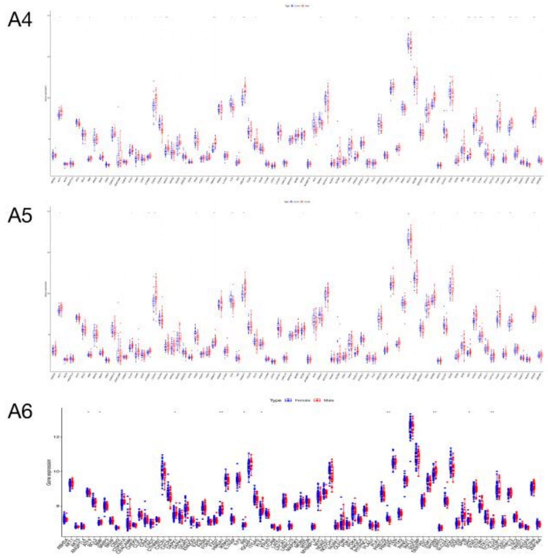

A4: Violin plot of ARDEGs between male samples and control samples.
A5: Violin plot of ARDEGs between female samples and control samples.
A6: Violin plot of ARDEGs between male samples and female samples.

FIG.S14 A1-A3 The heat map of male and female pyroptosis-related DEGs (PRDEGs)

FIG.S14

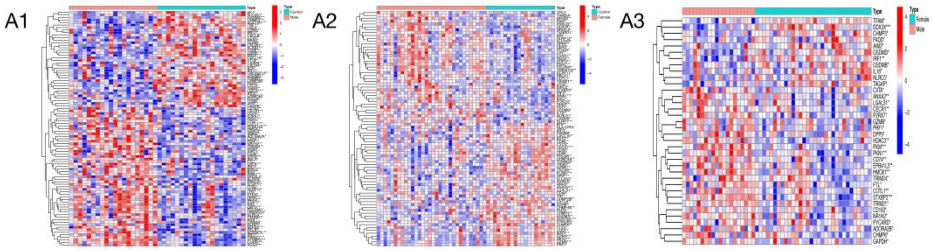

A1: The heat map of PRDEGs between male samples and control samples.
A2: The heat map of PRDEGs between female samples and control samples.
A3: The heat map of PRDEGs between male samples and female samples.

FIG.S 14A4-A6 Violin plot of male and female pyroptosis-related DEGs (PRDEGs)

FIG.S14

A4

A5

A6

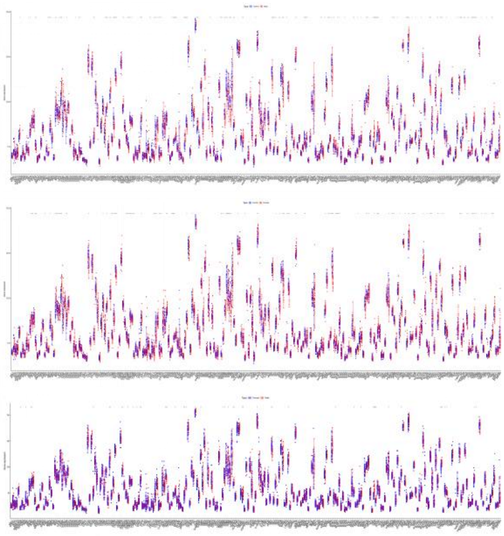

A4: Violin plot of PRDEGs between male samples and control samples.

A5: Violin plot of PRDEGs between female samples and control samples.

A6: Violin plot of PRDEGs between male samples and female samples.

FIG.S15 Machine learning algorithm for cell death-related DEGs (CDRDEGs) biomarkers between male samples and control samples

FIG.S15

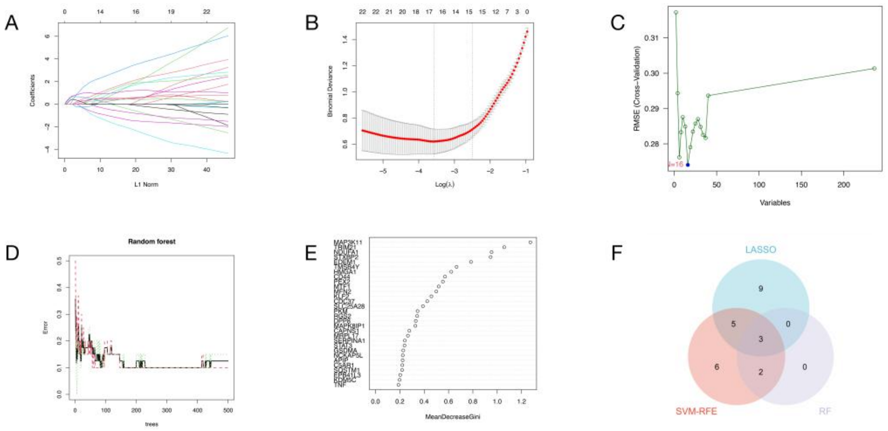

A: Ten iterations of cross validation were performed to adjust parameter selection in the LASSO model. Each curve represents a different gene.

B: Analysis of LASSO coefficients with a vertical dotted line indicating the optimal lambda value.

C: The SVM-RFE algorithm is used to select feature genes.

D: The error rate is influenced by the number of trees in the random forest.

E: Determining the significance of different genes in order of importance.

F: The Venn diagram illustrates the common genes identified by the LASSO, random forest, and SVM-RFE algorithms.

FIG.S16 Machine learning algorithm for cell death-related DEGs (CDRDEGs) biomarkers between female samples and control samples

FIG.S16

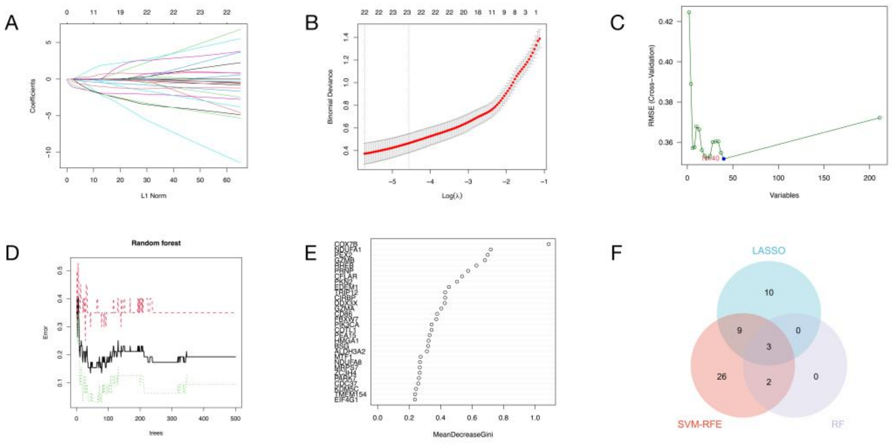

A: Ten iterations of cross validation were performed to adjust parameter selection in the LASSO model. Each curve represents a different gene.

B: Analysis of LASSO coefficients with a vertical dotted line indicating the optimal lambda value.

C: The SVM-RFE algorithm is used to select feature genes.

D: The error rate is influenced by the number of trees in the random forest.

E: Determining the significance of different genes in order of importance.

F: The Venn diagram illustrates the common genes identified by the LASSO, random forest, and SVM-RFE algorithms.

FIG.S17 Machine learning algorithm for cell death-related DEGs (CDRDEGs) biomarkers between male samples and female samples

FIG.S17

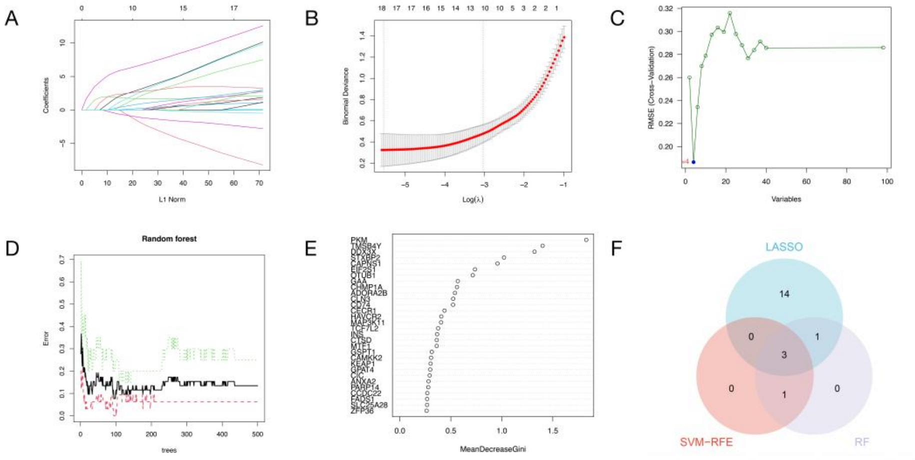

A: Ten iterations of cross validation were performed to adjust parameter selection in the LASSO model. Each curve represents a different gene.

B: Analysis of LASSO coefficients with a vertical dotted line indicating the optimal lambda value.

C: The SVM-RFE algorithm is used to select feature genes.

D: The error rate is influenced by the number of trees in the random forest.

E: Determining the significance of different genes in order of importance.

F: The Venn diagram illustrates the common genes identified by the LASSO, random forest, and SVM-RFE algorithms.

FIG.S18 Expression of EDEM1,MAP311,TRIM21 between male and control samples

FIG.S18

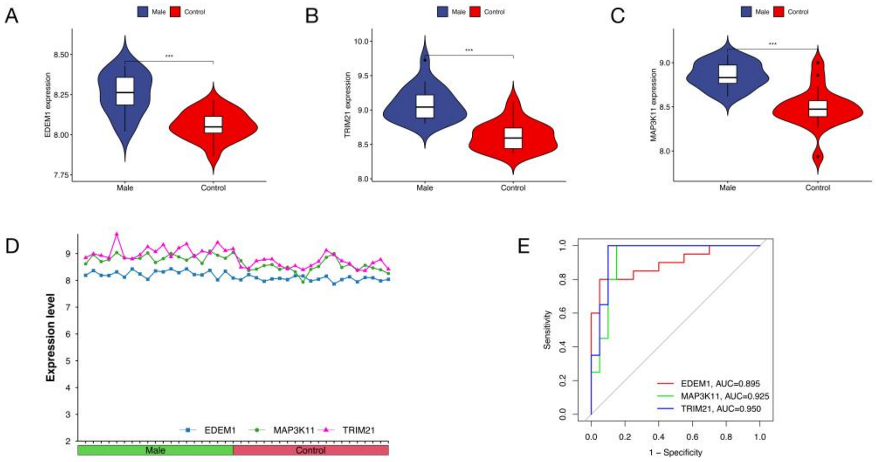

A-C: A violin map of the expression levels of three key genes in the two groups , EDEM1,MAP311 and TRIM21 were highly expressed in male samples.

D: The line chart the expression trend the three key genes in the two groups , EDEM1,MAP311 and TRIM21 were highly expressed in male samples.

E: ROC plots of three key genes in the two groups, The AUC of EDEM1,MAP311 and TRIM21 is 0.895,0.925 and 0.950.

FIG.S19 Expression of COX7B,PEX2,RHEB between female and control samples

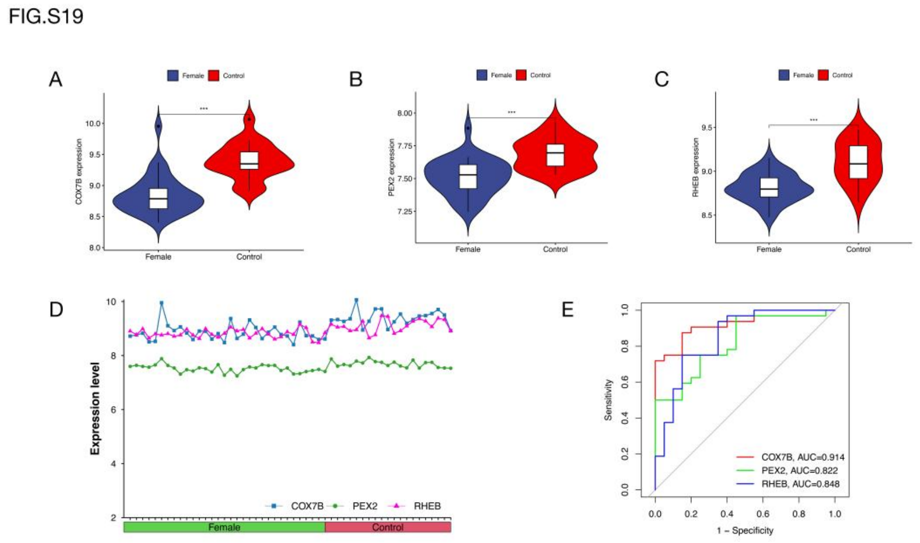

A-C: A violin map of the expression levels of three key genes between female and control samples, COX7B,PEX2,RHEB were low expressed in female samples.

D: The line chart the expression trend the three key genes between female and control samples, COX7B,PEX2,RHEB were low expressed in female samples.

E: ROC plots of three key genes between female and control samples, The AUC of COX7B,PEX2 and RHEB is 0.914,0.822 and 0.848.

FIG.S20 Expression of CAPNS1,DDX3X,TMSB4Y between male and female samples

FIG.S20

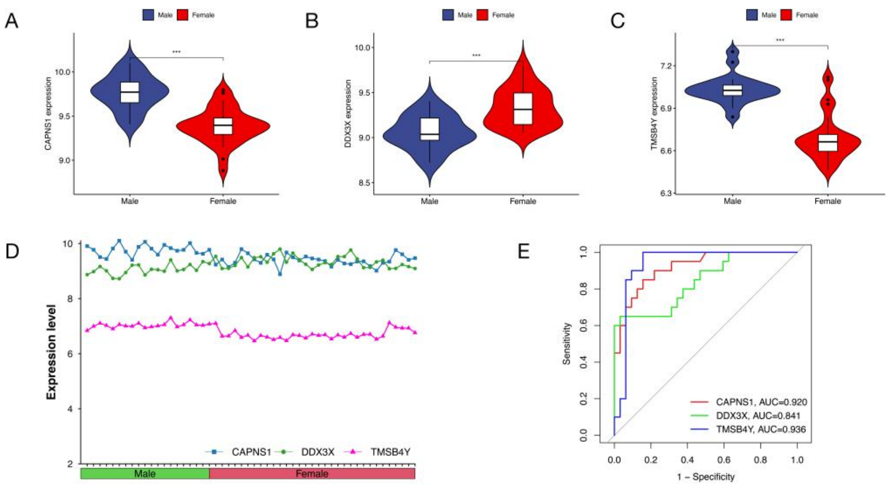

A-C: A violin map of the expression levels of three key genes between male and female samples, CAPNS1 and TMSB4Y are highly expressed in male samples, and DDX3X is low expressed in male samples.

D: The line chart the expression trend the three key genes between male and female samples, CAPNS1 and TMSB4Y are highly expressed in male samples, and DDX3X is low expressed in male samples.

E: ROC plots of three key genes between male and female samples,The AUC of CAPNS1,DDX3X and TMSB4Y is 0.920,0.841 and 0.936.

FIG.S21 The GSEA diagram of three key genes between male patient samples and control samples (A1 B1 C1 is the high expression group, A2 B2 C2 is the low expression group).

FIG.S21

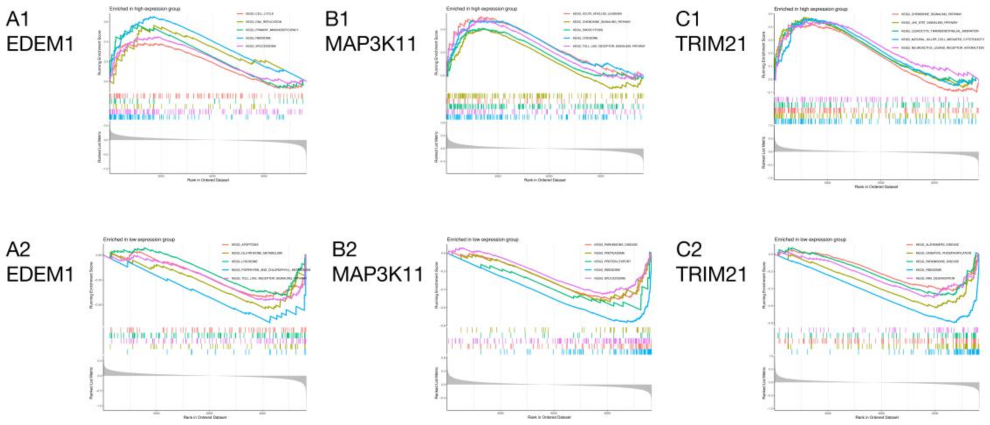

FIG.S22 The GSEA diagram of three key genes between female patient samples and control samples (A1 B1 C1 is high expression group, A2 B2 C2 is low expression group).

FIG.S22

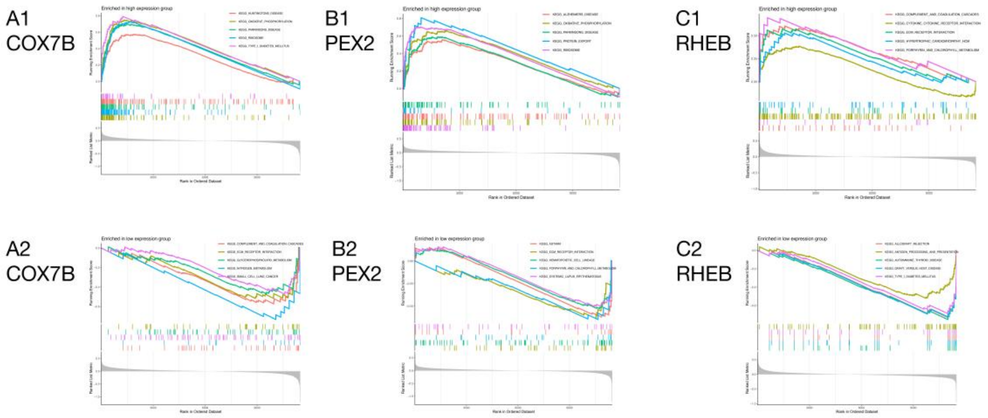

FIG.S23 The GSEA diagram of three key genes between male patient samples and female patient samples (A1 B1 C1 is the high expression group, A2 B2 C2 is the low expression group).

FIG.S23

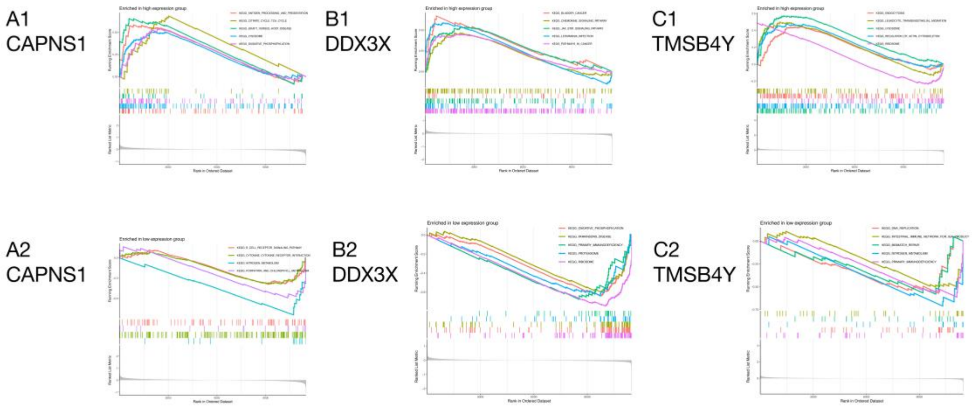

FIG.S24 A-B Immune correlation of EDEM1 and MAP3K11 in three groups.

FIG.S24

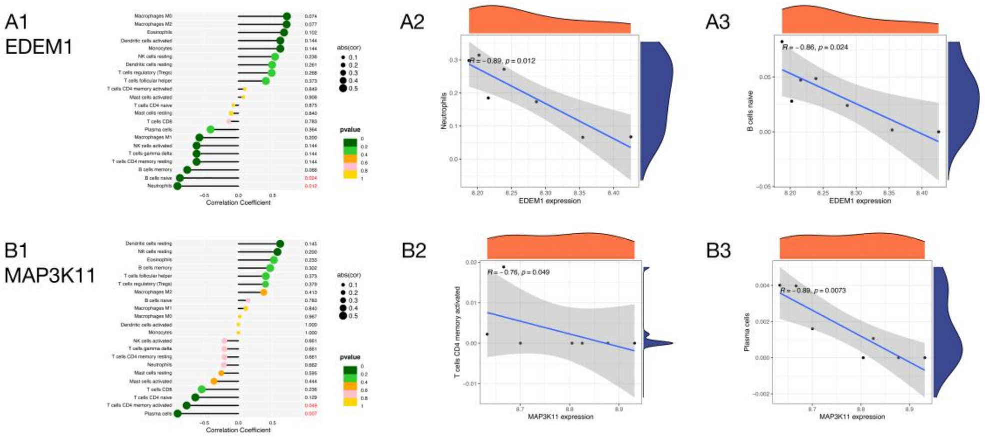

A : The immune correlation of EDEM1

B : The immune correlation of MAP3K11

FIG.S24C The immune correlation of TRIM21

FIG.S24

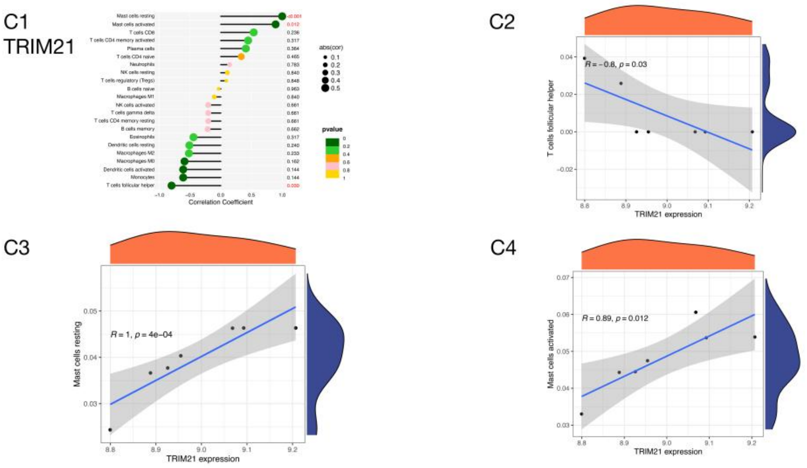

FIG.S24 D-E Immune correlation of COX7Band PEX2 in three groups.

FIG.S24

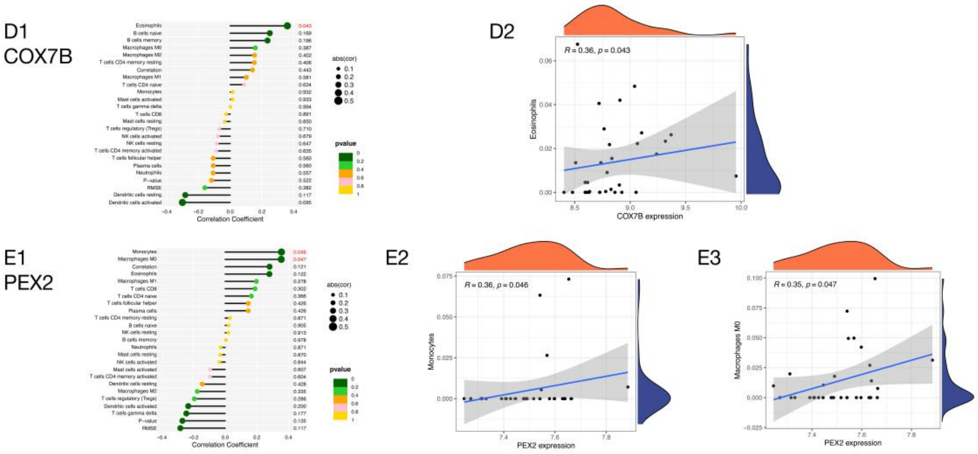

D: The immune correlation of COX7B

E: The immune correlation of PEX2

FIG.S24F The immune correlation of RHEB

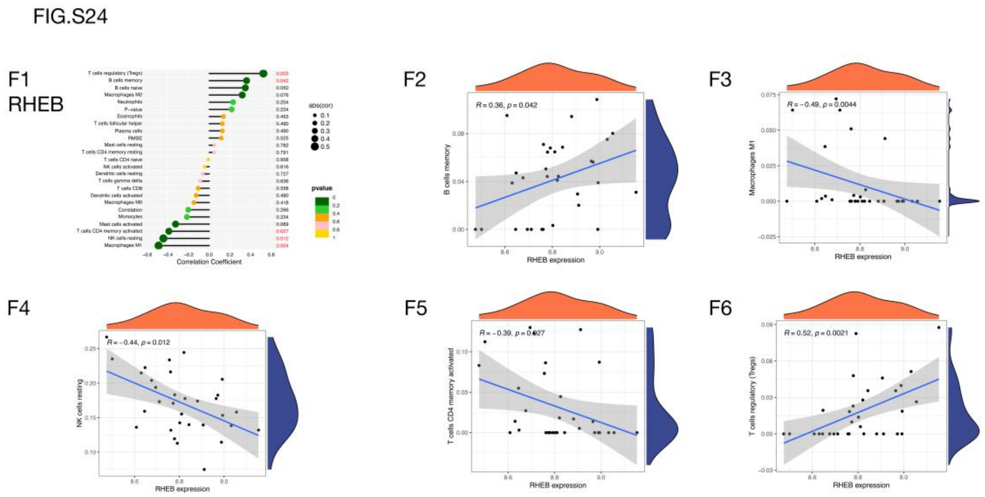

FIG.S24G The immune correlation of CAPNS1

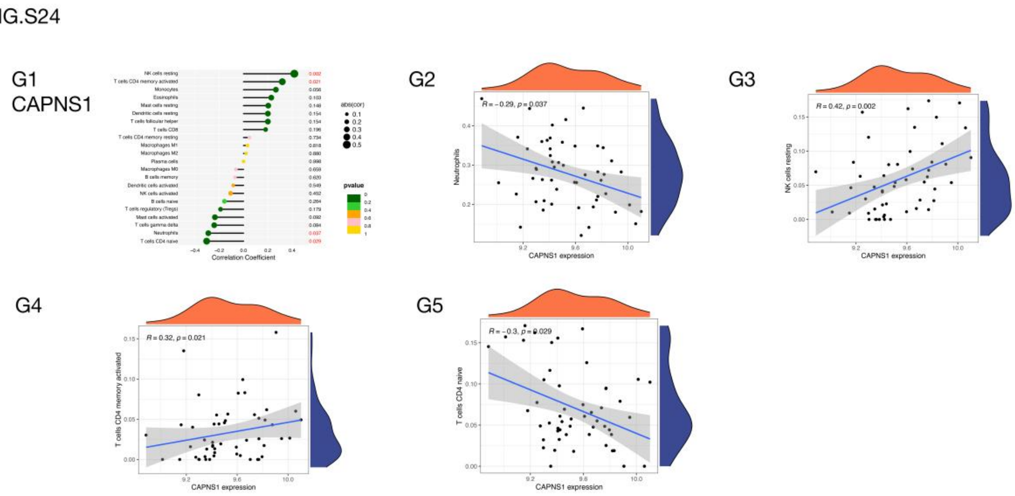

FIG.S24H The immune correlation of DDX3X

FIG.S24

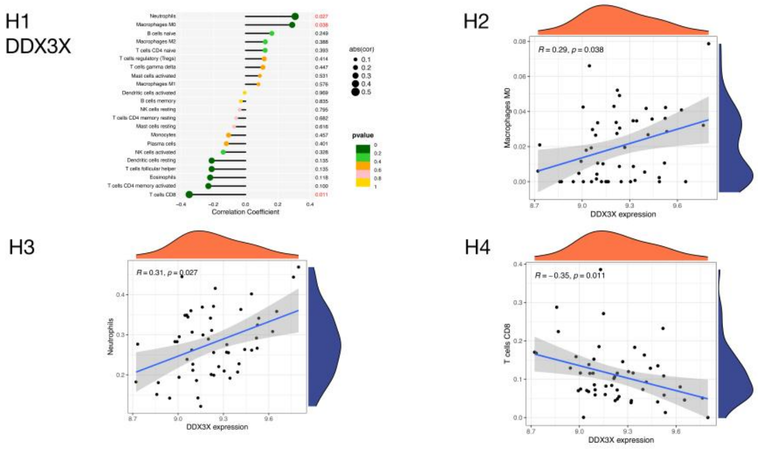

FIG.S24I The immune correlation of TMSB4Y

FIG.S24

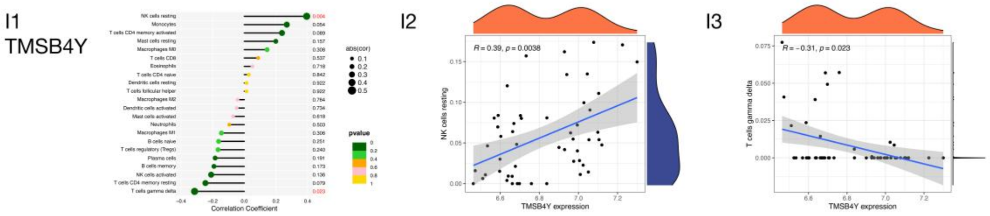

Supplement: Supplementary file 1 — Supplementary Figures. [file 41598_2024_65745_MOESM1_ESM.pdf]
